# Mass balance, metabolism, and pharmacokinetics of [^14^C]amdizalisib, a clinical-stage novel oral selective PI3Kδ inhibitor for the treatment of non-hodgkin’s lymphoma, in healthy Chinese volunteers

**DOI:** 10.3389/fphar.2024.1478234

**Published:** 2024-11-15

**Authors:** Chun-Yang Zhao, Li-Jun Zhang, Chan Sun, Cheng-Yin Yu, Jian Wang, Yang Sai, Wei-Guo Su, Qian Chen, Wei Wang, Jing-Ying Jia, Gang-Yi Liu, Yan-Mei Liu

**Affiliations:** ^1^ Drug Clinical Trial Center, Shanghai Xuhui Central Hospital/Xuhui Hospital, Fudan University, Shanghai, China; ^2^ Shanghai Engineering Research Center of Phase I Clinical Research and Quality Consistency Evaluation for Drugs, Shanghai, China; ^3^ Shanghai Institute of Materia Medica, Chinese Academy of Sciences, Shanghai, China; ^4^ HUTCHMED Limited, Shanghai, China

**Keywords:** amdizalisib, PI3Kδ kinase, metabolism, mass balance, metabolite identification, pharmacokinetics, safety

## Abstract

**Introduction:**

Amdizalisib (HMPL-689) is an ATP-competitive PI3Kδ inhibitor currently under investigation for treating Hodgkin’s lymphoma. This study aimed to evaluate the metabolism, excretion, pharmacokinetics, and safety profile of amdizalisib in healthy human subjects to support its clinical application.

**Methods:**

This Phase I clinical trial included six healthy Chinese male volunteers who received a single oral dose of 30 mg/100 µCi [^14^C]amdizalisib suspension. Blood, urine, and fecal samples were collected to analyze pharmacokinetics, metabolic pathways, and excretion patterns.

**Results:**

Amdizalisib was rapidly absorbed, with a median Tmax of 2.5 h. The C_max_ of 244 ± 48.9 ng/mL, and AUC_0-t_ was 1870 ± 474 h ng/mL after a single oral dose. The blood-to-plasma total radioactivity ratio ranged from 0.561 to 0.645, indicating no significant affinity of [^14^C]amdizalisib and its metabolites to blood cells and the radioactive material is mainly distributed in plasma. Excretion was primarily via feces and urine, with 62.08% ± 3.00% and 37.15% ± 2.84% of the dose recovered, respectively, and over 94% of the drug excreted within 96 h. The parent drug was the main radioactive component in plasma (51.45% of total radioactivity). Additionally, 11 metabolites were identified, and the metabolic pathways include oxidation on the benzene or pyrimidine rings and conjugation with cysteine or glucuronic acid. The major metabolites in plasma were the di-oxidized and hydrogenated product (M424) and the mono-oxidized product (M406-2), accounting for 16.67% and 20.91%, respectively. Both of them are also the major radioactive components in urine and feces, among of which M424 accounted for 21.01% and 14.26%, M406-2 accounted for 8.08% and 11.30%, of the administered dose in urine and feces, respectively. In addition, the di-oxidized and methylated product (M436) was one of the major metabolites in feces accounting for 17.7% of the administered dose. Few of the parent drug was found in urine and feces, suggesting primary metabolized in the liver. No serious adverse events or drug-related deaths occured, with diarrhea as the most common adverse event.

**Discussion:**

These findings demonstrate that amdizalisib is rapidly absorbed, extensively metabolized, and primarily excreted via feces and urine, supporting its continued development as a potential therapeutic for Hodgkin's lymphoma.

**Systematic Review Registration:**

https://www.chinadrugtrials.org.cn/, identifier CTR20212448.

## Introduction

Type I phosphoinositide 3-kinases (PI3Ks) are a group of lipid kinases consisting of four isoforms: α, β, δ and γ (Brown et al., 2014). Among them, PI3Kδ plays a critical role in the development and function of B lymphocytes. Aberrant activation of the PI3Kδ signaling pathway is closely associated with B-cell malignancies, making PI3Kδ inhibition an important therapeutic strategy for the treatment of B-cell non-Hodgkin lymphoma (NHL) (Lannutti et al., 2011; Hoellenriegel et al., 2011).

Amdizalisib is a highly selective and potent PI3Kδ inhibitor (Lawrence et al., 2020). Preliminary clinical studies have indicated antitumor activity of amdizalisib in susceptible tumors, alongside a manageable safety profile (Cohen et al., 2019). In pre-clinical studies, amdizalisib showed a good pharmacokinetic profile with good oral absorption, low clearance, extensive tissue distribution, low drug-drug interaction risk, and also showed good safety profile (Jiang et al., 2024). A phase I study assessing the food effect in healthy subjects in China has revealed that food intake could delay the oral absorption of amdizalisib, significantly prolong T_max_ and reduce C_max_, however, it had no obvious effect on the extent of absorption, as indicated by AUC. Meanwhile, it was showed that amdizalisib had a good safety profile in healthy subjects after a single dose, and no serious adverse reaction greater than grade 3 occurred (Cao W. et al., 2023).

Despite the promising antitumor activity and favorable pharmacokinetic properties observed in these studies, a comprehensive understanding of amdizalisib’s absorption, distribution, metabolism, and excretion (ADME) characteristics in human is still necessary. Specifically, elucidating the mass balance, metabolic pathways, and clearance mechanisms of this drug in human is crucial to support its ongoing clinical development (Ramamoorthy et al., 2022; Cerny et al., 2023). Radiolabeled mass balance studies, particularly those using [^14^C]-labeled compounds, are considered the “gold standard” (von Richter et al., 2016) for such investigations. These studies provide comprehensive data on drug recovery, metabolite disposition, and excretion routes, which are key to fully understanding the pharmacokinetic profile of a drug (Prakash et al., 2019; He et al., 2022; Coppola et al., 2019).

This study used [^14^C]amdizalisib in healthy Chinese adult male subjects to conduct a mass balance study aimed at determining the pharmacokinetics of amdizalisib in human, tracking its metabolic and excretory pathways, quantifying radioactivity distribution in blood and plasma, and assessing its clearance from the body. Furthermore, considering the potential for amdizalisib to be used in combination with other therapies, understanding its metabolism will help reduce the risk of drug-drug interactions and provide a basis for establishing appropriate clinical dosing regimens. This study is crucial for bridging the gap between preclinical data and clinical application.

## Materials and methods

### Study design

Amdizalisib, a small-molecule inhibitor of PI3Kδ kinase, is a patent product developed exclusively by HUTCHMED Limited. [^14^C]Amdizalisib dosing formulation was prepared by Value Pharmaceutical Services Co., LTD., lot number 20210929. This is a single-center, single-dose, non-randomized, and open-label clinical trial conducted in healthy Chinese male subjects, at Xuhui Central Hospital, Fudan University, Shanghai, China. The dose level is 30 mg/100 µCi.

This study strictly followed the scientific and ethical principles of the Declaration of Helsinki and Good Clinical Practice guidelines. Approval was granted by the ethics committee of Xuhui Central Hospital. All participants provided written informed consent before enrollment. The trial was registered on www.chinadrugtrials.org.cn (ID: CTR20212448).

Six subjects were admitted to the clinical research center 1 day prior to dosing, with random urine and fecal samples collected as blank excretion controls. After fasting overnight for at least 10 h, a blank blood sample was collected after a regular breakfast (449 kcal, with ∼35% energy from fat) on Day 1. Approximately 30 min post-breakfast, subjects received a single oral dose of [^14^C]amdizalisib suspension (30 mg containing 100 µCi radioactivity), which was prepared on the dosing day by adding ultrapure water into the [^14^C]amdizalisib oral dosing formulation, followed by a standardized meal at 4 h post-dosing. It is noteworthy that the medication administration duration for each subject was strictly kept within 10 min. Scheduled collection of urine, feces, and blood samples, and safety monitoring proceeded until completion.

The excreta sample collection ceased when total radioactivity from excreta surpassed 80% of the administered dose, or less than 1% of the dose for two consecutive intervals. Blood sample collection ceased when the radioactivity of plasma at 2 consecutive time points was lower than 3 times the radioactivity at 0 h before administration. Based on the interim results, sample collection concluded earlier than initially planned.

Venous blood samples were collected within 0.5 h prior to administration and at 0.5, 1, 1.5, 2, 3, 4, 6, 8, 12, 24, 36, 48, 72, and 96 h after administration. About 8 mL of blood was collected at each time point in the anticoagulant collection tube containing K_2_-EDTA. The centrifuged plasma was used for non-radioactive and radioactive pharmacokinetic and biotransformation (metabolite) analyses. In addition, about 2 mL of venous blood was collected within 0.5 h before administration and at 1, 4, 8, 12, 24, and 48 h after administration for whole blood radioactivity analysis. Additional 10 mL of venous blood for each time point was collected at 1, 4, 8, 12, 24, and 48 h after administration, and the plasma was obtained by centrifugation for biotransformation (metabolites) analysis. Urine and fecal samples were collected until 144 h (4 subjects) or 192 h (2 subjects) after administration.

### Study participants

Eligible participants were healthy males, aged 18–40, with normal bowel movements (1–2/day), body weight ≥50 kg, and BMI between 19–26 kg/m^2^. Health status was confirmed by medical history, vital signs, physical examination, 12-lead ECG, chest X-ray, and laboratory tests. Males of reproductive potential agreed to use effective contraception and refrain from sperm donation from admission until 180 days post-study.

Exclusion criteria were comprehensive, including significant medical history, impaired renal function, gastrointestinal surgery affecting drug absorption, severe allergies, prolonged QTcF on ECG, conditions affecting drug metabolism, substance abuse, recent blood donation, or exposure to radiation, among others.

### Pharmacokinetics

Plasma concentrations of amdizalisib were quantified using liquid chromatography-tandem mass spectrometry (LC-MS/MS). As the amdizalisib plasma quantitative LC-MS/MS method only detects the unlabeled analyte ([^12^C]amdizalisib) in plasma samples, a correction factor (1.022) obtained by the specific activity of the [^14^C]amdizalisib oral dose formulation, was entered into Analyst Data Processing Software (version 1.62) as a dilution factor. [^12^C]Amdizalisib in samples was conducted using a 5500QTRAP mass spectrometer coupled with a Shimadzu HPLC 20-AD ultra-fast liquid chromatography system. Chromatographic separation was achieved on an XBridge C18 column, maintained at 40°C, with a mobile phase consisting of 2 mM ammonium formate in water (A) and methanol (B). A gradient elution was employed at a flow rate of 0.6 mL/min. The autosampler was set to 5°C, with an injection volume of 5 μL. Detection was performed in multiple reaction monitoring (MRM) mode, tracking the transition of 391.2 m/z → 256.2 m/z for [^12^C]Amdizalisib. Each analytical run included calibration standards and quality control samples to ensure method accuracy and precision. The corrected total concentration data of labelled (^14^C) and unlabeled (^12^C) analytes were given directly by Analyst data processing software. The standard curve consists of 0.500, 1.00, 2.00, 10.0, 50.0, 200, 450, 500 ng/mL. The quality control (QC) sample concentrations were 0.5 (lower limit of quantification QC), 1.50 (lower QC), 20.0 (low to medium QC), 250 (medium QC), 400 (high QC) ng/mL, respectively.

Pharmacokinetic analysis was performed using Phoenix WinNonlin^®^ software version 8.3. The total plasma radioactivity concentration and unlabeled amdizalisib concentration at each blood collection time point were analyzed with descriptive statistics, and the statistical parameters included the number of cases, mean, standard deviation, coefficient of variation, median, quartile, minimum, maximum, geometric mean, and geometric coefficient of variation. Non-compartmental model was used to estimate the main PK parameters. Descriptive statistics applied to parameters including maximum plasma concentration after administration (C_max_), the time to reach maximum plasma concentration after administration (T_max_), elimination rate constant (λ_z_), area under the plasma concentration-time curve from the time 0 to the time of last quantifiable concentration (AUC_0-t_, obtained by the linear trapezoidal method), area under the plasma concentration-time curve from time 0 extrapolated to infinity (AUC_0-∞_, AUC_0-∞_ = AUC_0-t_ + C_t_/*λ*_*z*_), half-life (t_1/2_, t_1/2_ = ln (Lannutti et al., 2011)/λ_z_), mean residence time (MRT, MRT = AUMC/AUC), apparent clearance (CL/F, CL/F = Dose/AUC_0-∞_), apparent volume of distribution based on terminal phase (V_z_/F, V_z_/F = Dose/(λ_z_×AUC_0-∞_)).

### Excretion of radioactivity in urine and feces

Radioactivity was measured using an oxidative combustion apparatus and liquid scintillation counter (LSC). After the whole blood sample was vortexed thoroughly, two portions (about 0.5 g each) were weighed into the combustion boat, and after complete combustion by oxidative combustion apparatus, [^14^C]carbon dioxide generated from the combustion of the samples was captured by scintillation solution and measured by liquid scintillation counter. After plasma samples were fully vortexed, one portion (approximately 0.5 g) was weighed into a scintillation vial, mixed well with the scintillation solution, and measured using a liquid scintillation counter. When the radioactivity of the plasma sample after administration is below the LLOQ, additional samples can be added, up to a maximum of 1 g. After mixing the urine well, weighed one portion (about 1 g) into a scintillation bottle, then added scintillation solution to mix well, and use a liquid scintillation counter to measure the radioactivity. The feces were transferred to labelled containers with known empty weights and soaked in an appropriate amount of 50% aqueous isopropanol solution for about 2–4 h or for a certain period of time (e.g., overnight) at 1°C–9°C. Before homogenization, the solution was made up to a suitable dilution by the addition of an appropriate amount of 50% aqueous isopropanol, the total weight was weighed and recorded, and the homogenization was carried out using a tissue homogenizer. Two equal portions of the homogenate (approximately 0.3 g) were weighed into a combustion boat and fully combusted by an oxidative combustion apparatus, and the [^14^C]carbon dioxide generated from the combustion of the samples was captured with a scintillation solution and measured using a liquid scintillation counter.

The combustion efficiency of the oxidative combustor was verified by performing a combustion recovery test using a^14^C-containing standard solution of known radioactivity concentration prior to combustion of the sample. Oxidative combustion recoveries were derived by comparing the intensity of radioactivity measured by the liquid scintillation counter after complete combustion of the standard samples with that measured by the liquid scintillation counter after direct addition to the scintillation solution. The radioactivity concentration (disintegration per minute, DPM) of all biological samples measured on that day was divided by a correction factor to obtain the actual radioactivity intensity of the samples.

The ratio of total radioactivity in whole blood to plasma at each time point (K_b/p_) was calculated for each subject at each time point, along with the mean and standard deviation of the ratio at each time point.

### Metabolite profiling and identification

#### Plasma

The plasma samples from six subjects were pooled individually according to the AUC method to obtain 6 plasma samples of 0–72 h (Hop et al., 1998). A portion of the proportionally mixed plasma sample was taken and added to a certain volume of acetonitrile (3×, v/v) for extraction, vortexed for 1 min, shaken for 10 min and then stored in a refrigerator at 4°C for 30 min, the mixture was removed and vortexed for 2 min and then centrifuged for 10 min at 10,000×g and 4°C. The residue from the extraction was firstly ultrasonically suspended using 1 mL of ultrapure water and then extracted using methanol (2×, v/v) and repeated twice. The organic solvent extracts were combined, blown dry with nitrogen and reconstituted with acetonitrile: ultrapure water (1:1), the reconstituted solution was centrifuged at 4°C and 10,000×g for 10 min, and the supernatant was used for radio-metabolite profiling.

The radioactivity in the organic solvent extract and the reconstitution solution was determined by LSC. The solid residue after extraction was dissolved by heating with 1N KOH solution and added to the scintillation solution to determine its radioactivity. All the final data were used to examine the recovery of radioactivity during plasma extraction.

#### Urine

The urine samples from the six subjects were mixed in the same volume percentage to obtain six 0–72 h urine samples. A portion of the proportionally mixed urine sample was centrifuged for 10 min at approximately 10,000×g and 4°C. Two samples were taken before and after centrifugation, and the radioactivity of the samples was determined by LSC, and the data obtained were used to calculate the recovery of radioactivity during the centrifugation process. A portion of the supernatant after centrifugation was used directly for radio-metabolite profiling.

#### Feces

The fecal samples from the six subjects were mixed with the same weight percentage to obtain 6 fecal homogenate samples of 0–168 h. A portion of the proportionally mixed fecal homogenate samples was taken and extracted by adding a volume of acetonitrile (3×, v/v), vortexed for 5 min at room temperature, sonicated for 5 min and then centrifuged for 10 min at 10,000 × g and 4°C. The residue of the extracted material was firstly sonicated and mixed using about 1 mL of ultrapure water before extracted using methanol (2×, v/v). The process was repeated twice and the organic extracts were combined, blown dry using nitrogen and then reconstituted with dimethyl sulfoxide: acetonitrile: ultrapure water (3:2:5), the reconstituted solution was centrifuged at 10,000×g at 4°C for 10 min and the supernatant was used for radio-metabolite profiling. LSC was used to determine the intensity of radioactivity contained in the organic solvent extract and in the compound solution. LSC was used to determine the intensity of radioactivity contained in the organic solvent extract and in the reconstituted solution. The solid residue after extraction is fully combusted by an oxidative combustor, and the [^14^C]carbon dioxide produced by combustion of the sample is captured with a scintillation solution to determine its radioactivity intensity. All final data were used to examine the extraction and recovery of radioactivity from fecal homogenate samples.

The overall recoveries of the extraction methods in plasma, urine and feces are 90.37%–98.00%, 94.76%–104.50% and 94.76%–104.43%, indicating that the radioactivity was efficiently extracted. A high performance liquid chromatograph coupled with an online isotope detector and mass spectrometry (LC/RAM/MS) was used for the identification of radioactive metabolite profiles. The HPLC method consisted of an HPLC system (Shimadzu UFLC-20A-Lite), a chromatographic column (ACE Excel 3 C_18_, 150 × 4.6 mm id), mobile phase A (0.4% aqueous formic acid, pH = 3.2 adjusted with ammonia) and mobile phase B (acetonitrile) at a flow rate of 0.7 mL/min. The gradient was 0% B for the 0–5 min, 20% B from 15 min to 30 min, 22% B from 32 to 40 min, and 70%–100% B from 60 to 72 min. The column temperature was 28°C. The HPLC streams were collected in Deepwell LumaPlateTM-96 well plate at the rate of 15 s/part using an automatic fraction collector, and the radioactivity contained in each fraction was determined by using a microplate detector. The radioactivity contained in each fraction was determined using a microplate detector, and the results were converted using ARC^®^ Convert and Evaluation software to obtain offline radio metabolite profiles. The radioactive metabolite spectrum of each sample was integrated, the peak area of each radioactive metabolite was obtained, the percentage of each radioactive peak in the metabolite spectrum was determined (%), and the percentage of main metabolites in the total radioactivity in the corresponding matrix was calculated. The main metabolites were then calculated as a percentage of the total plasma radiation exposure (%AUC = Percentage of [^14^C]HMPL-689 and its metabolites in a radio-chromatogram of a plasma sample×the extraction yield of the sample) or as a percentage of the dose administered (%dose = the total amount of radioactivity detected in the sample (DPM)/The radioactivity administered (DPM)×100, urine and feces). All metabolite structure analyses were based on metabolite profiles, metabolic pathways, and HPLC relative retention time data, and the possible structure of each radioactive metabolite was inferred based on the molecular weights of the major radioactive peaks and the characteristics of their mass spectrum fragments.

### Safety and tolerability evaluation

The safety of the subjects was assessed by adverse events, laboratory test results, vital signs, and 12-lead electrocardiogram (ECG). AEs were recorded from the time of administration and serious adverse events (SAE) were recorded from the time that subjects signed the informed consent. Subjects with elevated aspartate aminotransferase (AST) and/or alanine aminotransferase (ALT) levels (≥3 × ULN) combined with elevated serum total bilirubin (≥2 × ULN) or clinical jaundice in the same blood sample should be recorded as a specific adverse event. Adverse events were coded using the Medical Dictionary (MedDRA, 24.1).

### Statistical analysis

Phoenix WinNonlin^®^ (version 8.3, Certara) was used to perform a descriptive statistical analysis of the plasma total radioactive concentration and the concentration of amdizalisib at each blood collection time point. Statistical parameters include N, mean, standard deviation, coefficient of variation, median, interquartile range, minimum, maximum, geometric mean, geometric coefficient of variation, etc. The total radioactivity ratio (K_b/p_) in whole blood and plasma at each time point was calculated for each subject, as well as the mean value and standard deviation of the ratio at each time point. Below the lower limit of quantification (BLQ) samples of whole blood or plasma are treated as missing when calculating the individual ratio, and were not involved in the calculation of the mean value in that case. According to the actual dose of the subjects, the radioactive concentration and weight data of excreta, the percentage of total radioactive excreta to the dose, the cumulative excretion rate and the total recovery rate were calculated at each time interval. The descriptive statistical summary of the mean and standard deviation of the excretion rate data was carried out, and the cumulative excretion rate curve was plotted. When urine and fecal radioactivity concentrations are BLQ, zero value will be used in the calculation of the recovery rate. The number of AEs and the percentage of treatment-emergent adverse events (TEAE) were summarized by System Organ Class (SOC) and/or Preferred Term (PT) and/or Common Terminology Criteria for Adverse Events (CTCAE) grade.

## Results

### Participants’ distribution and baseline characteristics

Six subjects were successfully enrolled (Table 1). All enrolled subjects successfully completed the study. The mean (standard deviation) age, weight and BMI were 29.0 (5.10) years, 65.32 (4.376) kg and 22.43 (1.405), respectively (See Table 1).

**TABLE 1:** Demographics and baseline disease characteristics.

Parameter		Total (N = 6)
Age, years	Mean (SD)	29.0 (5.10)
	Median (range)	26.5 (25, 36)
Gender	Male	6
Race	Asian	6
Height (cm)	Mean (SD)	170.67 (3.869)
Weight (kg)	Mean (SD)	65.32 (4.376)
BMI (kg/m^2^)	Mean (SD)	22.43 (1.405)
Smoking history, n%	Never smoked	6
Drinking history, n%	Not drinking regularly^a^	6

BMI, body mass index; SD, standard deviation.

^a^ Drinking regularly was defined as drinking more than 21 units of alcohol per week (1 unit = 360 mL of beer or 45 mL of 40% spirits or 150 mL of wine).

### Pharmacokinetics

**FIGURE 1:**
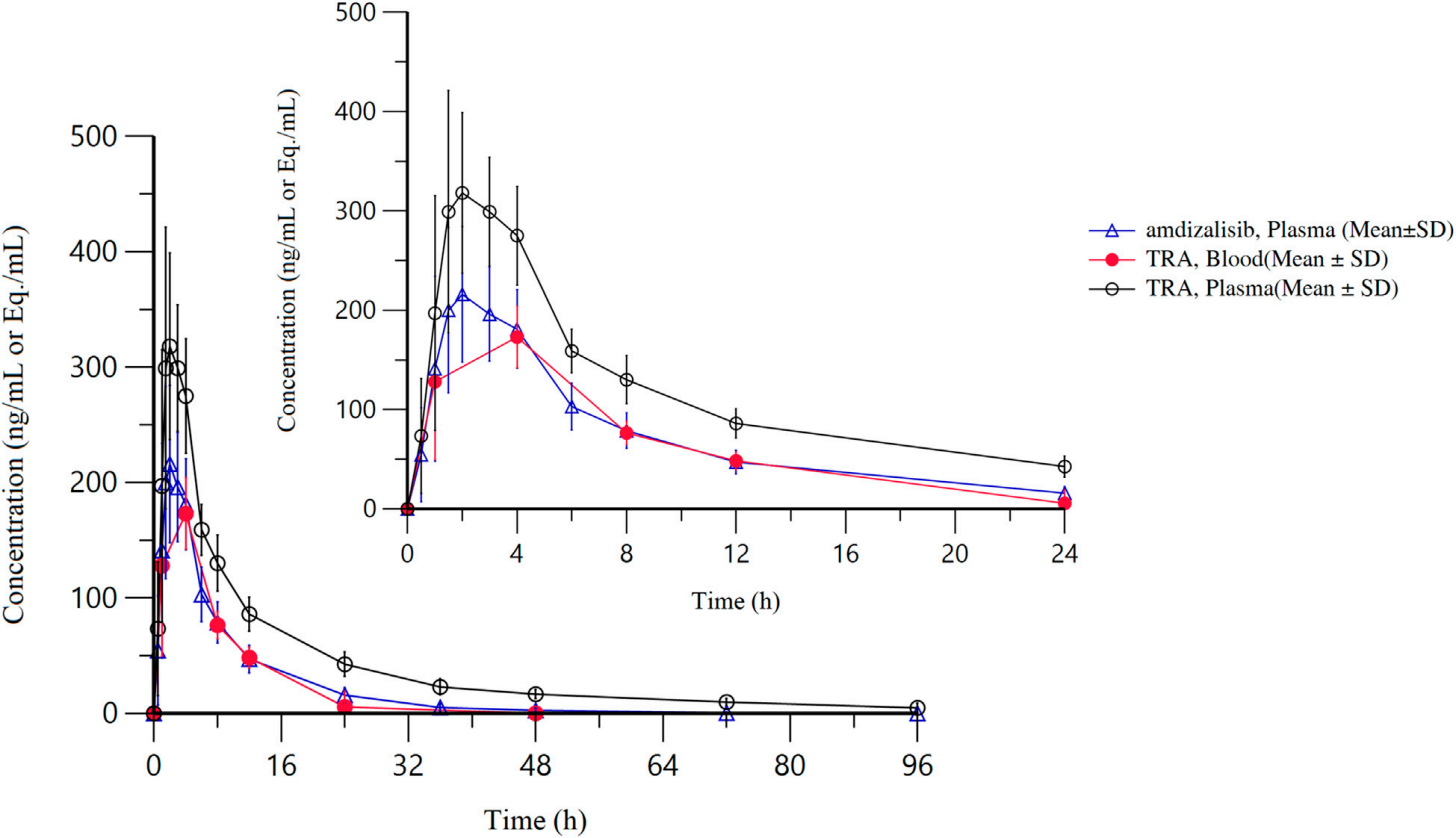
The mean concentration-time curves of total radioactivity and amdizalisib after a single oral administration of 30 mg/100 µCi [^14^C]amdizalisib suspension in 6 healthy Chinese males (linear coordinate).

**FIGURE 2:**
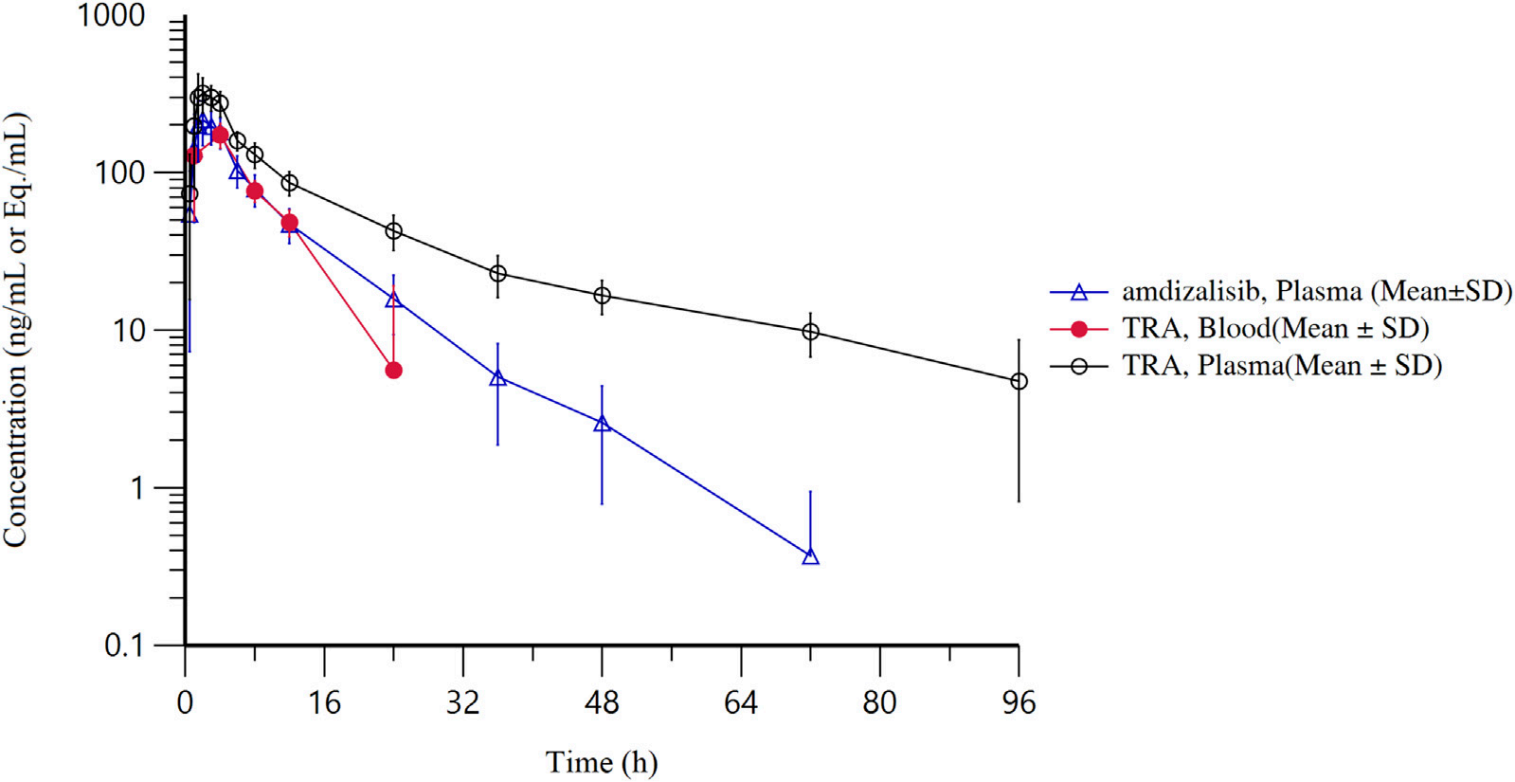
The mean concentration-time curves of total radioactivity and amdizalisib after a single oral administration of 30 mg/100 µCi [^14^C]amdizalisib suspension in 6 healthy Chinese males (semi-logarithmic coordinate).

After a single oral administration of [^14^C]amdizalisib suspension, the mean concentration-time curves of amdizalisib and total radioactivity in blood and plasma show that the total radioactivity in blood is lower than that in plasma at each time point (see Figure 1 for linear coordinates; Figure 2 for semi-logarithmic coordinates). The median T_max_ of amdizalisib in plasma was 2.50 h, and the arithmetic mean of C_max_ was 244 ng/mL. At 72 h post dose, the plasma concentration of amdizalisib could be detected in only 2 subjects, and was below the lower quantifiable limit (0.500 ng/mL) in the other 4 subjects. The arithmetic mean of AUC_0-t_ was 1870 h·ng/mL, MRT_0-t_ was 9.65 h, estimated t_1/2_ was 8.34 h, V_z_/F was 192 L, and CL/F was 16.8 L/h (See Table 2).

**TABLE 2:** Pharmacokinetic parameters of total radioactivity and Amdizalisib in plasma after a single oral administration of 30 mg/100 µCi [^14^C] Amdizalisib suspension to 6 healthy Chinese males (means ± SD).

Parameter	Total radioactivity (N = 6)	Amdizalisib (N = 6)
^a^ *T*_max_	2.50 (1.50,4.00) h	2.50 (1.50,4.00) h
*C*_max_	368 ± 63.0 ng Eq./mL	244 ± 48.9 ng/mL
AUC_0-t_	3,900 ± 647 h ng Eq./mL	1870 ± 474 h ng/mL
AUC_0-∞_	4,240 ± 711 h ng Eq./mL	1880 ± 478 h ng/mL
MRT_0-t_	18.8 ± 2.46 h	9.65 ± 1.48 h
*t*_*1/2*_	32.3 ± 7.83 h	8.34 ± 2.43 h
V_z_/F	337 ± 94.5 L	192 ± 28.0 L
CL/F	7.25 ± 1.30 L/h	16.8 ± 4.44 L/h

AUC_0-t_, area under the serum concentration–time curve from time 0 to the time of last quantifiable concentration; AUC_0-∞_, area under the serum concentration–time curve from time 0 extrapolated to infinity; CL, total clearance; *C*_max_, maximum observed serum concentration after administration; *T*_max_, time to maximum observed serum concentration after administration; *t*_*1/2*_, half-life; Vz/F, apparent volume of distribution. AUC_0-∞_ = AUC_0-t_ + C_last_/*λ*_*z*_.

^a^ *T*_*max*_ is presented as median (minimum, maximum).

The median T_max_ of total plasma radioactivity (equivalent molar of amdizalisib) was 2.50 h, similar to that of the parent drug, with the arithmetic mean C_max_ of 368 ng Eq./mL. A small amount of radioactivity was still detectable in the plasma of 4 subjects at 96 h post dose, with an average concentration of 4.74 ng Eq./mL. The arithmetic mean of AUC_0-t_ for total radioactivity in plasma was 3,900 h·ng Eq./mL, MRT_0-t_ was 18.8 h, the estimated average t_1/2_ was 32.3 h, V_z_/F was 337 L, and CL/F was 7.25 L/h (See Table 2). The total radioactivity ratio of whole blood to plasma of quantifiable samples within 24 h after administration was less than 1, and the average ratio at different time points was between 0.561 and 0.645.

### Excretion of radioactivity in urine and feces

**FIGURE 3:**
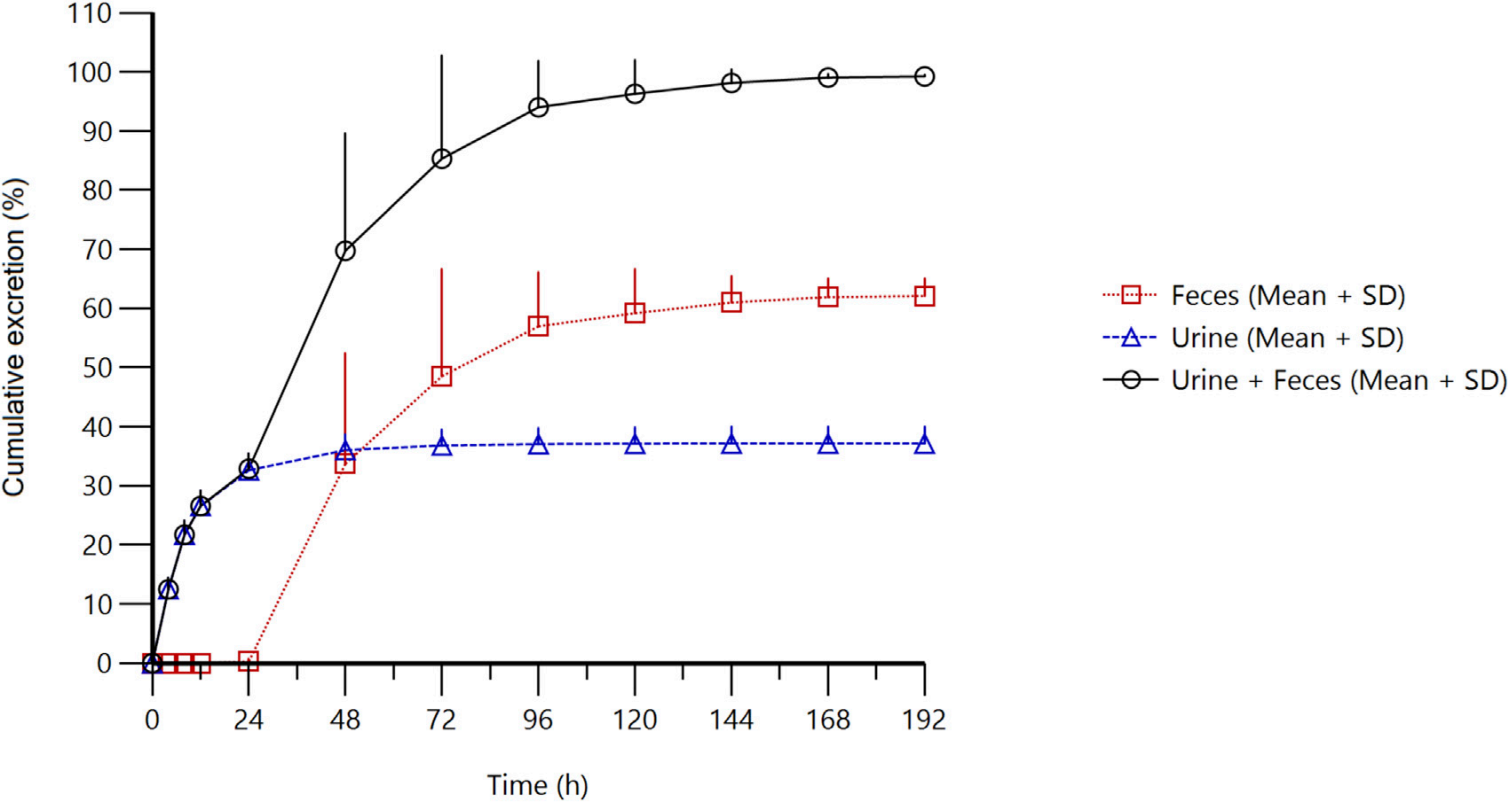
Cumulative excretion of radioactivity in urine and feces from subjects receiving a single oral administration of 30 mg/100 µCi [^14^C]amdizalisib suspension.

After a single oral administration of 30 mg/100 µCi [^14^C]amdizalisib suspension for 6 healthy male subjects, the percentage of total radioactivity recovered in urine and feces to the dosage was shown in Table 3, and the cumulative excretion rate curve was shown in Figure 3.

**TABLE 3:** Cumulative excretion of radioactivity in urine and feces after a single oral administration of 30 mg/100 µCi [^14^C] Amdizalisib suspension.

Parameter	Subject						Total (mean ± SD)
	1,001	1,002	1,003	1,004	1,005	1,006	
Ae_urine_, % of dose	40.40	35.85	35.12	40.65	37.11	33.74	37.15 ± 2.84
Ae_feces_, %of dose	58.49	63.33	64.48	58.70	61.77	65.73	62.08 ± 3.00
Ae_total_, % of dose	98.89	99.18	99.60	99.35	98.88	99.47	99.23 ± 0.30

Urine and feces were collected for 0–192 h from subjects 01,001 and 01,002, and 0–144 h from subjects 01,003 to 01,006.

The results showed that the total radioactivity recovered from urine and feces after administration accounted for 99.23% ± 0.30% of the dose, the excretion of radioactivity was 62.08% ± 3.00% in the feces and 37.15% ± 2.84% in urine. The excretion of total radioactivity mainly occurred within 96 h after administration, and the average excretion during this period accounted for 94.01% of the administered dose, and accounted for 94.74% of the total excretion from 0 h to 192 h.

### Metabolite profiling

**FIGURE 4:**
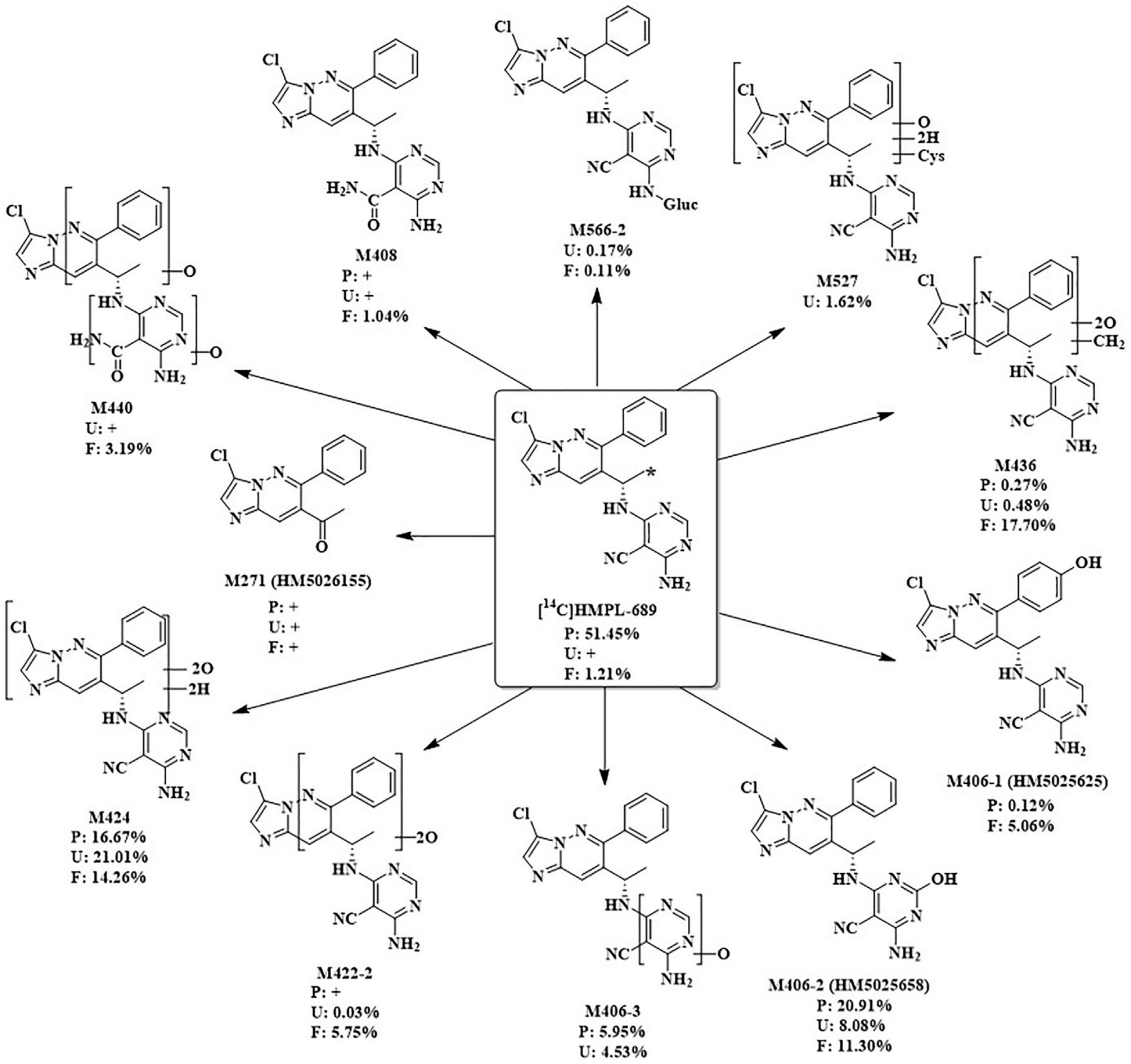
The proposed metabolic pathways of amdizalisib in healthy male subjects *: The position that ^14^C labelled. +: The metabolite was detected by mass spectrometry, but could not be quantified due to the low radioactivity %: %AUC in plasma, %Dose in Urine and feces.

**FIGURE 5:**
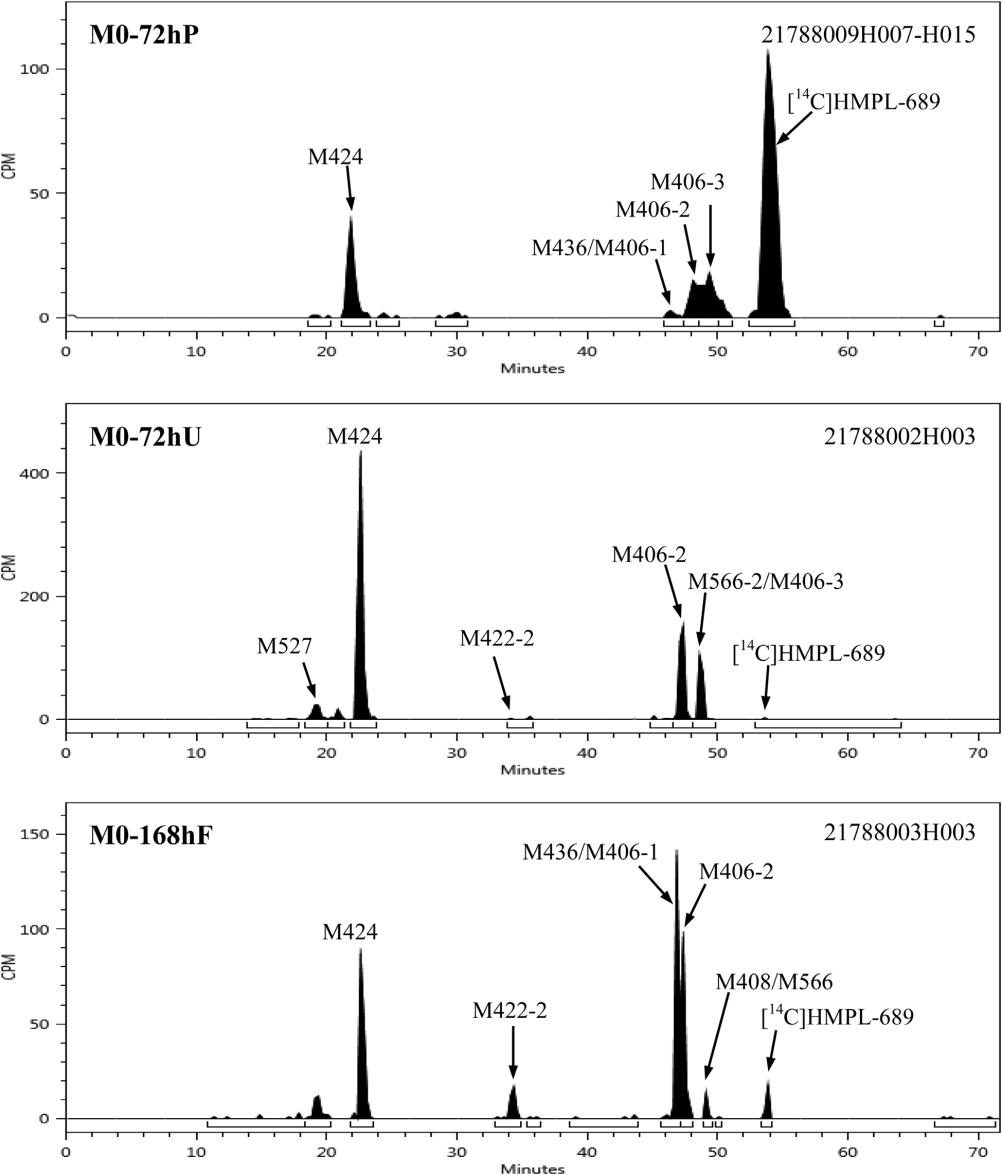
Representative radio-chromatograms of the metabolites in human plasma, urine, and fecal samples.

After a single oral administration of 30 mg/100 µCi [^14^C]amdizalisib suspension in 6 healthy male subjects, the structure of metabolites in human plasma, urine, and feces samples were identified using LC-MS/MS. In addition to the parent drug, 11 metabolites were identified. The main metabolic pathways are: 1) oxidation of benzene ring or pyrimidine ring; 2) cysteine or glucuronic acid conjugation. The proposed metabolic pathway for amdizalisib was shown in Figure 4. The metabolic results in plasma and excreta were shown in Table 4. The representative radio-chromatograms of pooled samples (0–72 h plasma, 0–72 h urine, and 0–168 h feces) from six subjects are shown in Figure 5, the individual radio-chromatograms of pooled 0–72 h plasma samples, 0–72 h urine samples, and 0–168 h fecal samples are shown in Supplementary Figures S12–S14. M436 and M406-1 were observed to co-elute in both plasma and feces samples, M406-3 and M566-2 co-eluted in urine samples, and M408 and M566-2 co-eluted in feces samples. The %AUC and %Dose values for these metabolites were calculated based on mass intensity, as shown in Supplementary Figures S15–S18.

**TABLE 4:** [14C]Amdizalisib and its main metabolites as percentage of total plasma radioactivity (%AUC) and percentage of dose in urine and feces (% dose).

Metabolites	The type of metabolites	Plasma	Urine	Feces
		%AUC	%Dose (37.15%)	%Dose (62.08%)
Amdizalisib	Parent drug	51.45	+	1.21
M527	Oxidation and cysteine conjugation	ND	1.62	ND
M440	Tri-oxidation and hydrogenation	ND	+	3.19
M424	Di-oxidation and hydrogenation	16.67	21.01	14.26
M422-2	Di-oxidation	+	0.03	5.75
M436	Di-oxidation and methylation	0.27	0.48	17.70
M406-1	Monooxidation, confirmed as HM5025625	0.12	ND	5.06
M406-2	Monooxidation, confirmed as HM5025658	20.91	8.08	11.30
M406-3	Monooxidation	5.95	4.53	ND
M566-2	Glucuronic acid conjugation	ND	0.17	0.11
M408	Oxidation and hydrogenation	+	+	1.04
M271	Oxidation and dealkylation, confirmed as HM5026155	+	+	+
Total identified peaks		95.37	35.92	59.62
Total unidentified peaks		4.63	1.23	2.46
Percentage of peaks identified		95.37	96.69	96.04

ND: not detectable

+: No radioactive peaks were detected, but mass spectrometry peaks were detected

The radioactive peaks identified in plasma accounted for 95.37% of the total radioactivity in circulating system and the representative chromatogram was shown in Figure 5. The parent drug was the main radioactive component in plasma. The main metabolites detected in plasma were M424 (16.67%) and M406-2 (20.91%) (See Table 4). The total radioactive exposure percentage (%AUC) of [^14^C] Amdizalisib and its metabolites in the plasma samples of individual subjects is shown in Supplementary Table S1, with individual differences in the parent drug and major metabolites. Other radioactive metabolites identified in plasma included M406-3 (5.95%), M436 (0.27%), and M406-1 (0.12%). In addition, some radioactive peaks were not identified, and no single radioactive peak was higher than 1.34% of the total radioactivity in plasma. In addition, the metabolites M422-2, M408, and M271 were detected by mass spectrometry, but could not be quantified due to their low radioactivity.

M424 (di-oxidation hydrogenation product): Analysis by LC/(+)ESI-FTMS revealed that the [M + H]⁺ of M424 was m/z 425.1240, corresponding to the molecular formula C_19_H_18_ClN_8_O_2_⁺. The primary characteristic fragment ions generated from the [M + H]⁺ collision are depicted in Supplementary Figure S1. Under the (+) HCD mode, the cleavage of the C-N bond in M424 results in fragment ions *m/z* 290 and *m/z* 136; the fragment ion *m/z* 290 undergoes further dehydration to produce the fragment ion *m/z* 272; the fragment ion *m/z* 272 then proceeds to break N-N and C-C bonds, forming the fragment ion *m/z* 170. The proposed fragmentation pathway for the characteristic properties of M424 is shown in Supplementary Figure S1.

M406-2 (mono-oxidation product): LC/(+)ESI-FTMS analysis showed that [M + H]^+^ produced by M406-2 was *m/z* 407.1134, which was consistent with the molecular formula C_19_H_16_ClN_8_O^+^. The main characteristic fragment ion spectra produced by the [M + H]^+^ collision are shown in Supplementary Figure S2.

M436 (di-oxidation and methylation product): LC/(+)ESI-FTMS analysis showed that [M + H]^+^ produced by M436 was *m/z* 437.1237, which was consistent with the molecular formula C_20_H_18_ClN_8_O_2_^+^. The main characteristic fragment ion spectra produced by [M + H]^+^ collisions are shown in Supplementary Figure S3. In the (+)HCD mode, the fragmentation pattern of M436 was basically consistent with that of amdizalisib standard. The putative characteristic spectral cleavage pathway of M436 is shown in Supplementary Figure S3. The putative characteristic spectral cleavage paths of other metabolites are shown in Supplementary Figure S4–S11.

The total radioactivity excreted by urine from 0 to 144/192 h was 37.15% of the dose. The parent drug was detected only by mass spectrometry. The main metabolite in urine was M424 (21.01%) (See Table 4). The percentage of Dose (%Dose) of [^14^C] Amdizalisib and Its Metabolites in Pooled Urine Samples for Individual Subjects is shown in Supplementary Table S2, with individual differences in the major metabolite. Other radioactive metabolites were M527 (1.62%), M406-2 (8.08%), M406-3 (4.53%), M422-2 (0.03%), M436 (0.48%), and M566-2 (0.17%). In addition, the metabolites M440, M408, and M271 were detected by mass spectrometry, but could not be quantified due to their low radioactivity.

Fecal excretion is one of the main excretion routes. The total radioactivity excreted by feces from 0 to 144/192 h after administration was 62.08% of the dose. Among them, the parent drug only accounted for 1.21% of the dose. The main metabolites in feces are M424 (14.26%), M436 (17.70%), and M406-2 (11.30%) (See Table 4). The percentage of Dose (%Dose) of [^14^C] Amdizalisib and Its Metabolites in Pooled Fecal Samples for Individual Subjects is shown in Supplementary Table S3, with individual differences in the parent drug and some metabolites. Other radioactive metabolites were M440 (3.19%), M422-2 (5.75%), M406-1 (5.06%), M566-2 (0.11%) and M408 (1.04%). In addition, the metabolite M271 was detected by mass spectrometry, but could not be quantified due to the low radioactivity.

### Safety and tolerability

A total of 5 subjects (83.3%) reported 11 TEAEs throughout the study, all of which were grade 1 in severity and were considered the study drug related. No subjects reported serious adverse events or adverse events leading to death. All TEAEs were in remission and recovered at the end of the study. There was no change in dosing schedule due to TEAE. During the study, four subjects reported TEAE as diarrhea (66.7%) and two subjects reported abnormal laboratory test results (33.3%) including shortened ECG PR, elevated low-density lipoprotein, and positive urine white blood cells. One subject received combination medication (Tylenol) and physical hypothermia for upper respiratory tract infection.

## Discussion

This radiolabeled mass balance study of amdizalisib provided a comprehensive analysis of its pharmacokinetic behavior and metabolic profiles in healthy male subjects, shedding light on the compound’s biodistribution and excretory pathways. The study findings are crucial for understanding the therapeutic potential and safety of amdizalisib, particularly in the context of its application in cancer therapy. Amdizalisib is utilized in cancer treatment, but its non-cytotoxic nature permits its evaluation in healthy volunteers (Karakunnel et al., 2018). Preclinical animal studies have shown that the drug has a good safety profile (Jiang et al., 2024), and a study assessing human food effects in healthy subjects, alongside a Phase I dose escalation and expansion study in cancer patients, have demonstrated tolerable safety levels. These studies consistently reported that any treatment-induced adverse events (TEAEs) were mild and transient, emphasizing the drug’s safety across diverse settings (Cao W. et al., 2023) (Cao J. et al., 2023). Conducting trials with healthy volunteers allows for an accurate assessment of amdizalisib’s pharmacokinetic properties without interference from underlying diseases or other medications. This methodology significantly enhances experimental control, thereby bolstering the reliability of the obtained data (Karakunnel et al., 2018).

In addition, all trials involving healthy subjects adhere to strict ethical standards and regulatory requirements. This particular study was approved by the Ethics Committee and only carried out with the informed consent of all participants to ensure that ethical integrity and participant safety are maintained at all times.

After a single oral dose of 30 mg/100 µCi [^14^C]amdizalisib suspension under fed condition in healthy Chinese male subjects, C_max_ and AUC_0-∞_ of amdizalisib in plasma was higher than those obtained from the previous food-effect study under fed condition (Cao W. et al., 2023) (C_max_: 244 ng/mL vs. 152 ng/mL; AUC_0-∞_: 1880 h٠ng/mL vs. 1,574 h٠ng/mL) in which Chinese healthy volunteers were dosed with 30 mg amdizalisib capsules. This may be due to the different drug formulations used but still need to be further explored whether the suspension formulation can improve the degree of oral absorption of amdizalisib. In addition, the variability among individuals and the differences in study conditions may also contribute to it. The pharmacokinetic data of amdizalisib showed that the median T_max_ of total plasma radioactivity and the parent drug were both 2.50 h, and then the concentration of the parent drug in plasma decreases significantly. This suggests a moderate rate of absorption and a rapid initial distribution phase. The discrepancy in the t_1/2_ between amdizalisib and its total radioactivity (8.34 h vs. 32.3 h) highlights extensive metabolic processing and possibly the retention of metabolites in less perfused tissues.

The total radioactivity ratios of whole blood to plasma of quantifiable samples were all less than 1, and the average ratio at different time points was between 0.561 and 0.645, indicating that there was no significant affinity of amdizalisib and its metabolites to blood cells and the radioactive material is mainly distributed in plasma.

A total of 11 metabolites were identified in plasma, urine, and stool samples. The main metabolic pathway *in vivo* is oxidation on benzene ring or pyrimidine ring, and conjugation with cysteine and glucuronic acid. The main radioactive component in plasma is the parent drug, accounting for 51.45% of the total plasma radioactivity. Two major metabolites including one di-oxidation and hydrogenation product (M424) and one mono-oxidation product (M406-2) were identified in the circulating system, accounting for 16.67% and 20.91% of the total plasma radioactivity, respectively.

Urine and feces are both major excretory pathways. The total recovery of radioactivity from urine and feces after drug administration accounted for 99.23% ± 0.30% of the administered dose, with a higher excretion in feces at 62.08% ± 3.00% and a lower excretion in urine at 37.15% ± 2.84%, which is similar to the data obtained from the preclinical radiolabeled mass balance study conducted in rats (Jiang et al., 2024). The excretion of total radioactivity primarily occurred within 96 h after drug administration indicating amdizalisib could be quickly excreted out of the human body. This potentially mitigated the risk of accumulation and related toxicity in patients with impaired renal or hepatic function.

In both urine and feces, the parent drug was not the major radioactive component, suggesting that amdizalisib is mainly metabolized by the liver in the human body. The main metabolite in urine was M424, accounting for 21.01% of the dose. The main metabolites in feces were M424, di-oxidation and methylation products (M436) and mono-oxidation metabolite (M406-2), accounting for 14.26%, 17.70%, and 11.30% of the dose, respectively. Although the radioactivity recovered in feces were higher than 60%, considering all of the metabolites observed in feces were oxidation metabolites mainly mediated by CYPs and few of parent drug was found, it can be deduced that the radioactivity in feces was probably sourced from the bile excretion but not from the metabolism in the intestines by microbiota. Therefore, the oral absorption of amdizalisib could be expected to be high.

According to the reaction phenotyping study using cDNA-expressed recombinant human cytochrome P450s and silensomes™ as well as human liver microsomes with selective chemical CYP inhibitors that was conducted in Hutch-Med DMPK department, CYP2C9 and aldehyde oxidase are the major enzymes involved in the metabolism of amdizalisib to M424 and M406-2, respectively (unpublished data). The enzymes that involved in another main metabolite, M436, found in feces, are still unknown. For other metabolites, it has been demonstrated that M406-1 was formed primarily by CYP2C9 and the formation of M406-3 was probably mediated by CYP3A4. Considering M406-2, M424 plus M406-1 contributed to around 20% and 40% of amdizalisib metabolism, respectively, CYP2C9 and AO could be confirmed to be the major metabolic enzymes responsible for amdizalisib metabolism. In addition, CYP3A4 also contributed to some extent. Phase II metabolic enzymes like UGTs showed very limited contribution to amdizalisib metabolism due to few of conjugated metabolites identified. Overall, multiple enzymes involved in the metabolism could help to mitigate the drug-drug interaction (DDI) risk of amdizalisib in human when co-administered with enzyme perpetrators.

The safety profile of the test drug, as observed in our study, is good and acceptable. The main TEAEs were diarrhea and fever, which were similar to those reported in the previous food effect study (Cao W. et al., 2023).

## Conclusion

After a single oral dose of [^14^C]Amdizalisib suspension, it could be absorbed quickly and completely excreted within 168 h, with both urine and feces as the main excretion routes. Oxidation is the predominant metabolic pathway and few of phase II conjugated metabolites were found. Few of the parent drug was recovered in the excreta, indicating the oral absorption of amdizalisib is good. The safety profile was manageable, and no new safety signals were noted during the study.

## Data Availability

The raw data supporting the conclusions of this article will be made available by the authors, without undue reservation.

## Supplementary material

SUPPLEMENTARY FIGURE 1 Mass spectra and the proposed fragmentation profiles of M424.

SUPPLEMENTARY FIGURE 2 Mass spectra and the proposed fragmentation profiles of M406-2.

SUPPLEMENTARY FIGURE 3 Mass spectra and the proposed fragmentation profiles of M436.

SUPPLEMENTARY FIGURE 4 Mass spectra and the proposed fragmentation profiles of M527.

SUPPLEMENTARY FIGURE 5 Mass spectra and the proposed fragmentation profiles of M440.

SUPPLEMENTARY FIGURE 6 Mass spectra and the proposed fragmentation profiles of M422-2.

SUPPLEMENTARY FIGURE 7 Mass spectra and the proposed fragmentation profiles of M406-1.

SUPPLEMENTARY FIGURE 8 Mass spectra and the proposed fragmentation profiles of M406-3.

SUPPLEMENTARY FIGURE 9 Mass spectra and the proposed fragmentation profiles of M408.

SUPPLEMENTARY FIGURE 10 Mass spectra and the proposed fragmentation profiles of M566-2.

SUPPLEMENTARY FIGURE 11 Mass spectra and the proposed fragmentation profiles of M271.

SUPPLEMENTARY FIGURE 12 Radioactive liquid chromatography profile of plasma samples from healthy male subjects 01001–01006 at 0–72 h.

SUPPLEMENTARY FIGURE 13 Radioactive liquid chromatography profile of urine samples from healthy male subjects 01001–01006 at 0–72 h.

SUPPLEMENTARY FIGURE 14 Radioactive liquid chromatography profile of fecal samples from healthy male subjects 01001–01006 at 0–168 h.

SUPPLEMENTARY FIGURE 15 Mass spectrogram of M436 and M406-1 co-effluents in a plasma sample.

SUPPLEMENTARY FIGURE 16 Mass spectrogram of M436 and M406-1 co-effluents in a fecal sample.

SUPPLEMENTARY FIGURE 17 Mass spectrogram of M406-3 and M566-2 co-effluents in a urine sample.

SUPPLEMENTARY FIGURE 18 Mass spectrogram of M408 and M566-2 co-effluents in a fecal sample.
