# Supplementary figures and images for: Mass balance, metabolism, and pharmacokinetics of [14C]amdizalisib, a clinical-stage novel oral selective PI3Kδ inhibitor for the treatment of non-hodgkin’s lymphoma, in healthy Chinese volunteers
Source: Front Pharmacol. 2024 Nov 15;15:1478234. doi: 10.3389/fphar.2024.1478234 (PMC11605291; doi:10.3389/fphar.2024.1478234)

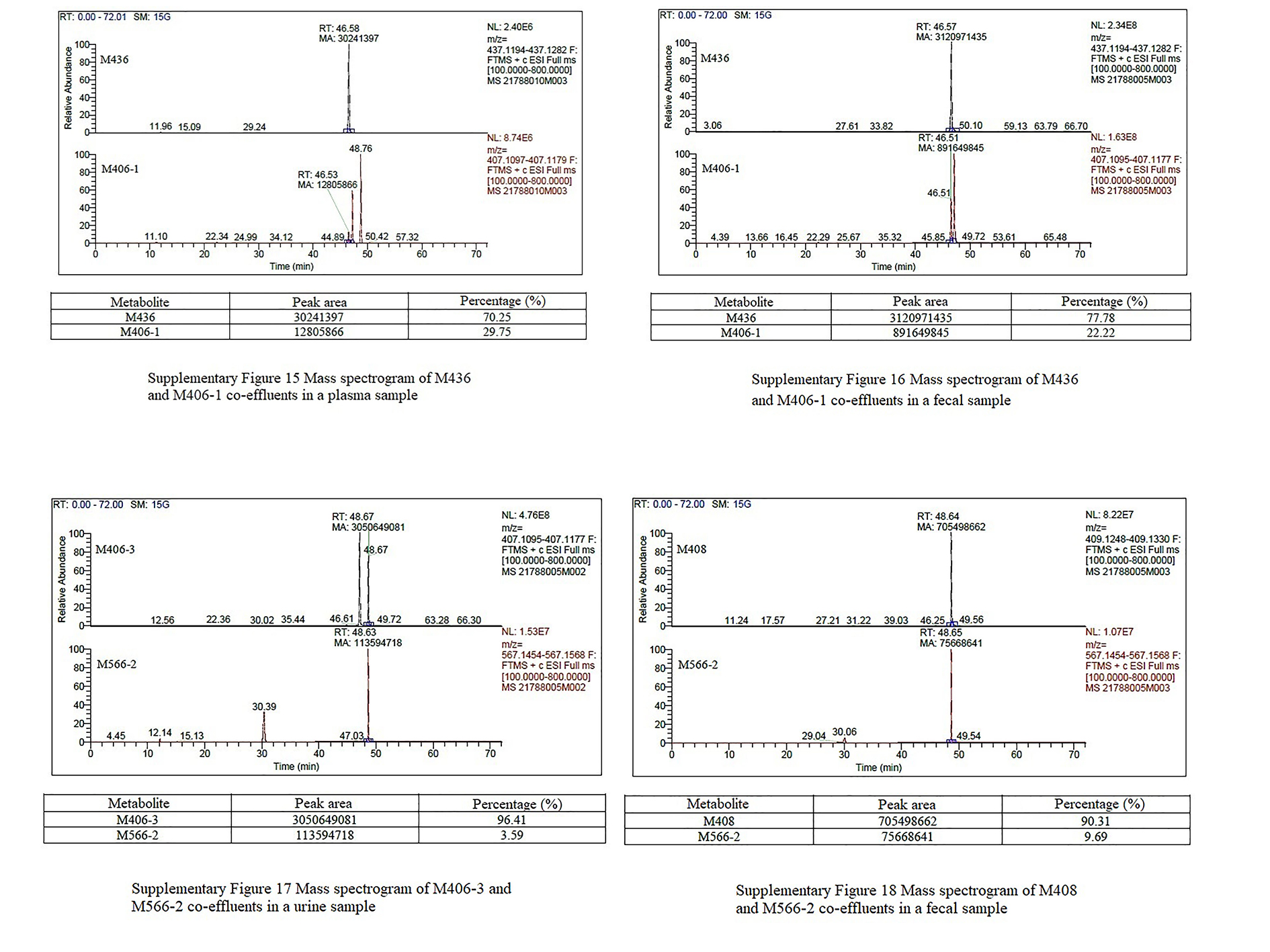

Supplement: Supplementary file 1 [file Image15.JPEG]

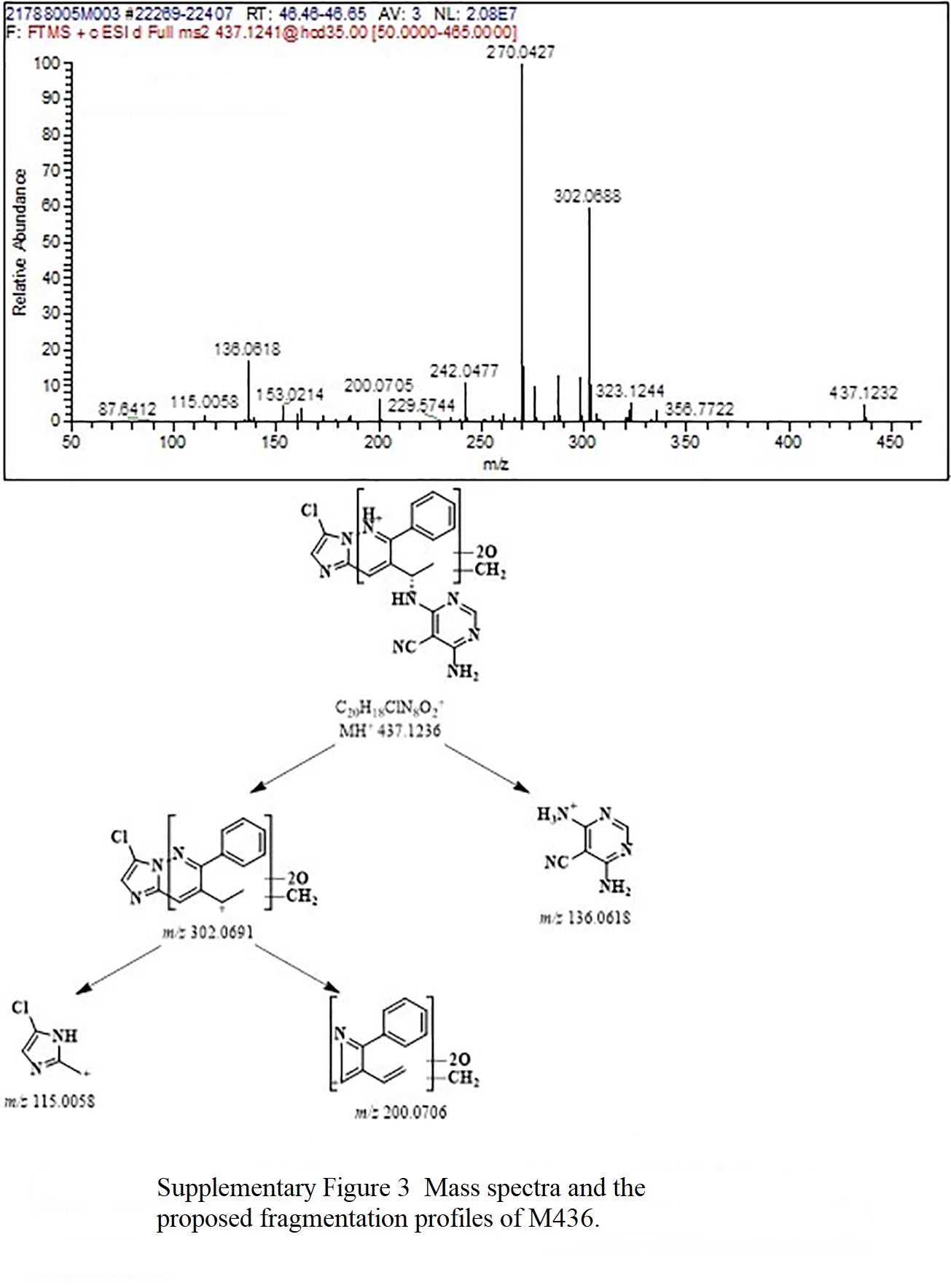

Supplement: Supplementary file 2 [file Image3.JPEG]

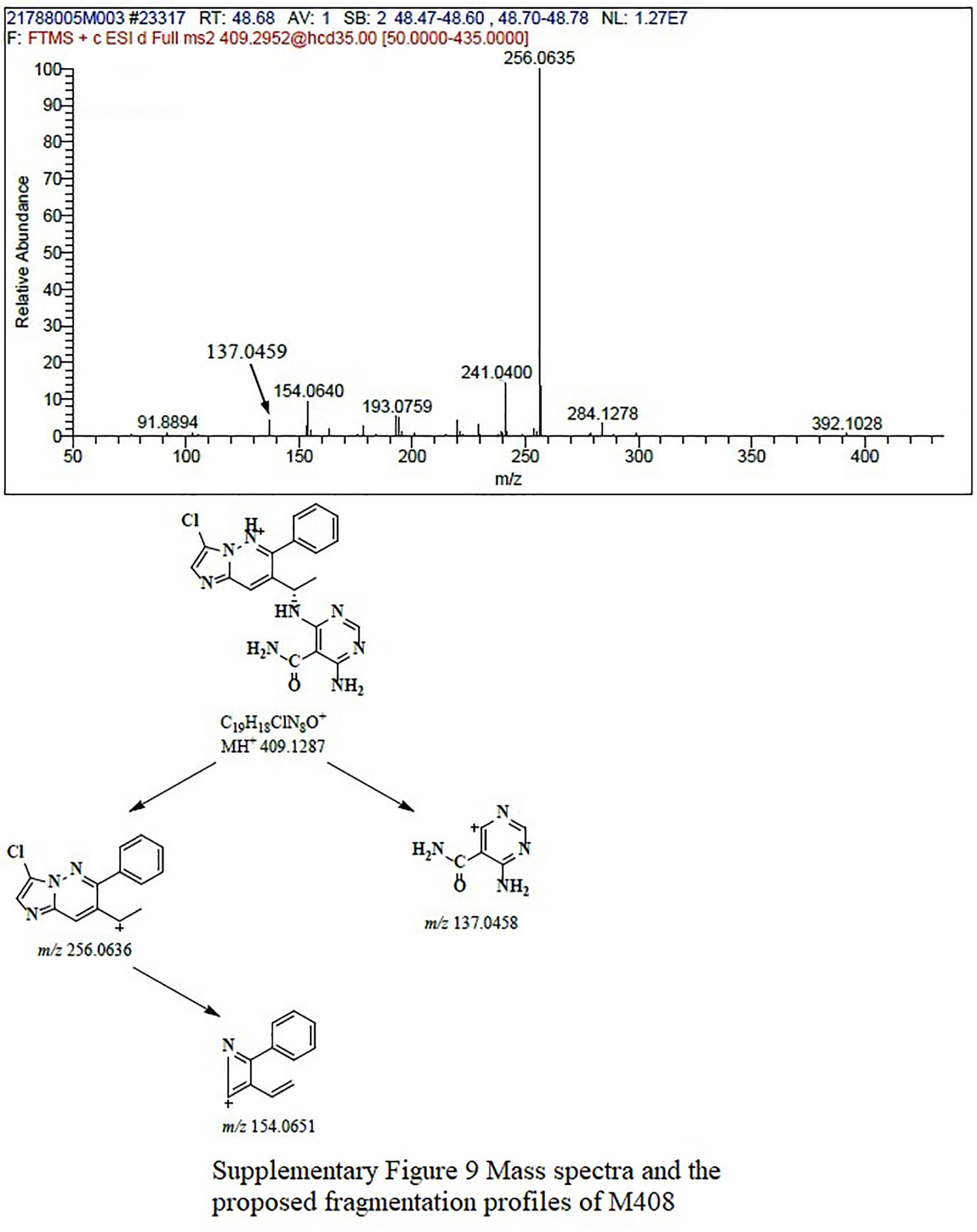

Supplement: Supplementary file 4 [file Image9.JPEG]

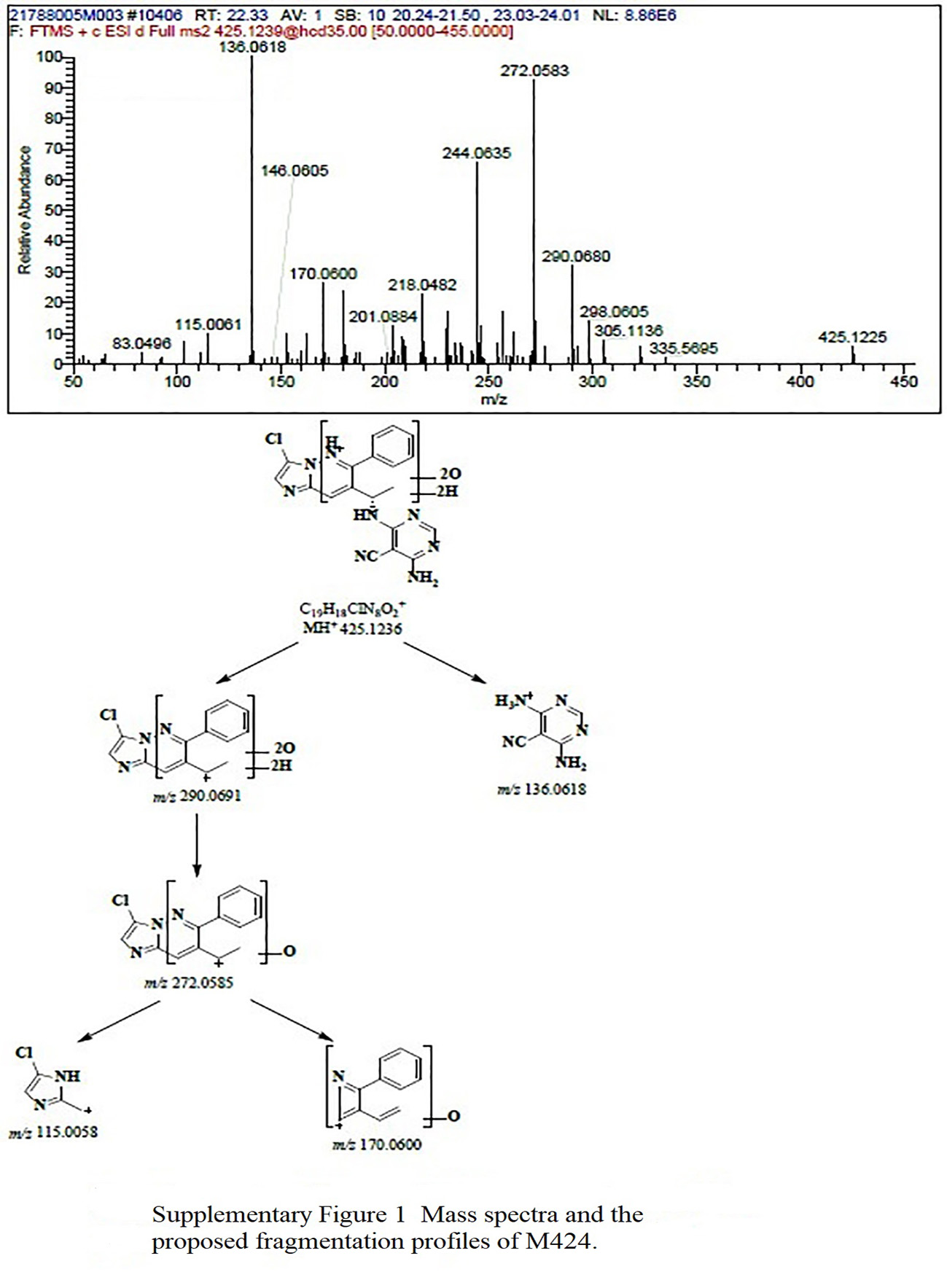

Supplement: Supplementary file 5 [file Image1.JPEG]

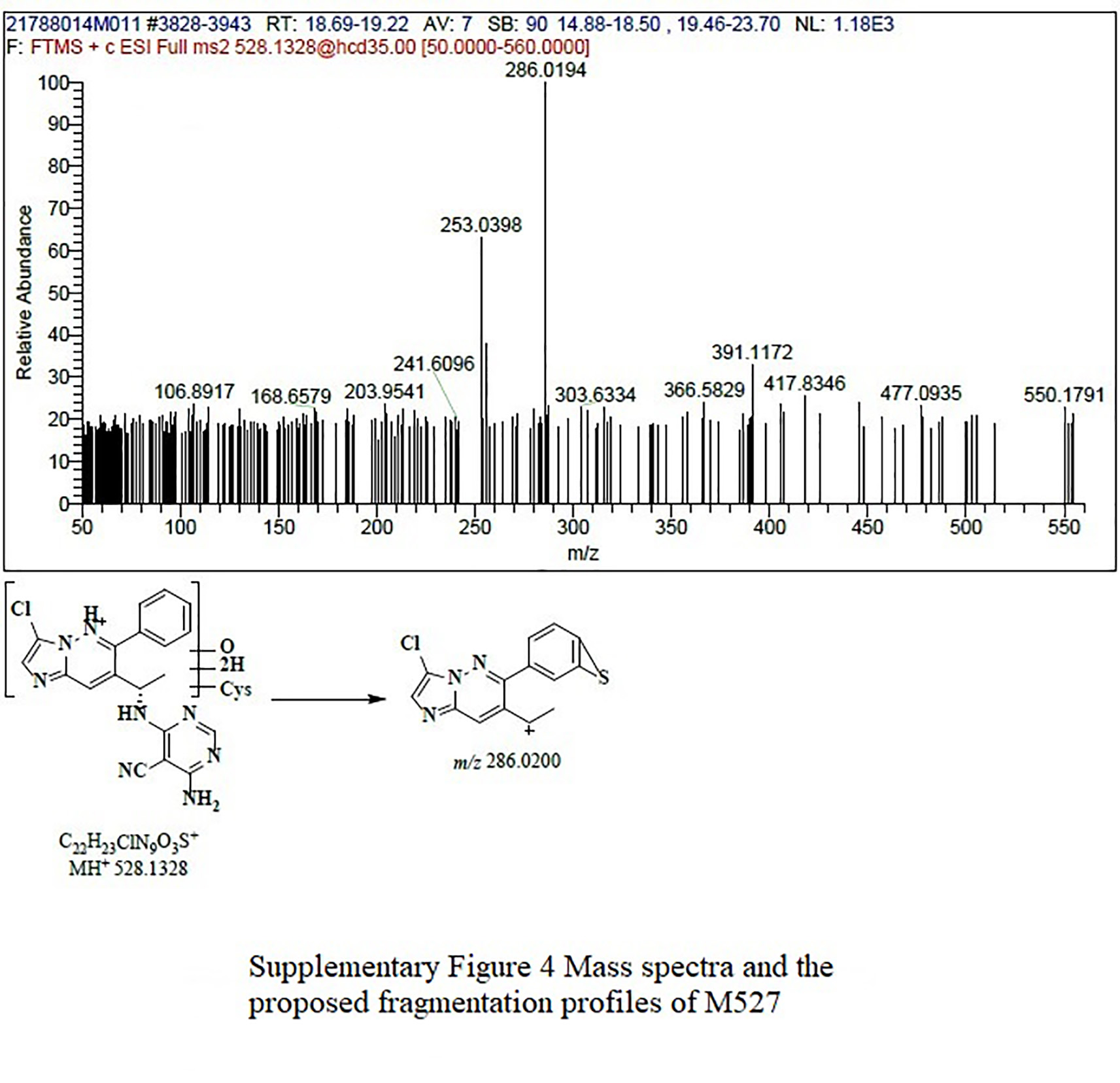

Supplement: Supplementary file 6 [file Image4.JPEG]

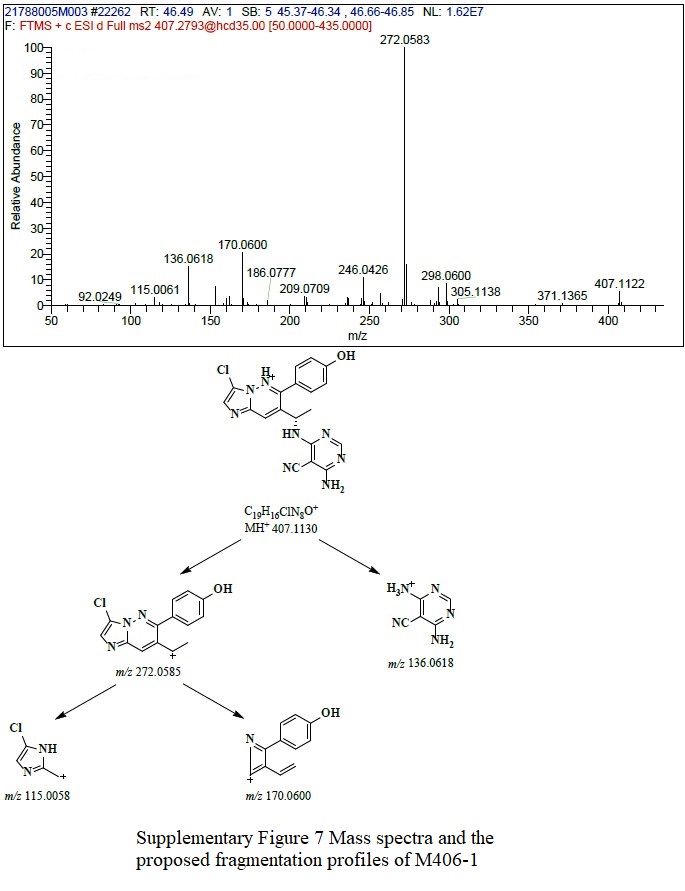

Supplement: Supplementary file 7 [file Image7.JPEG]

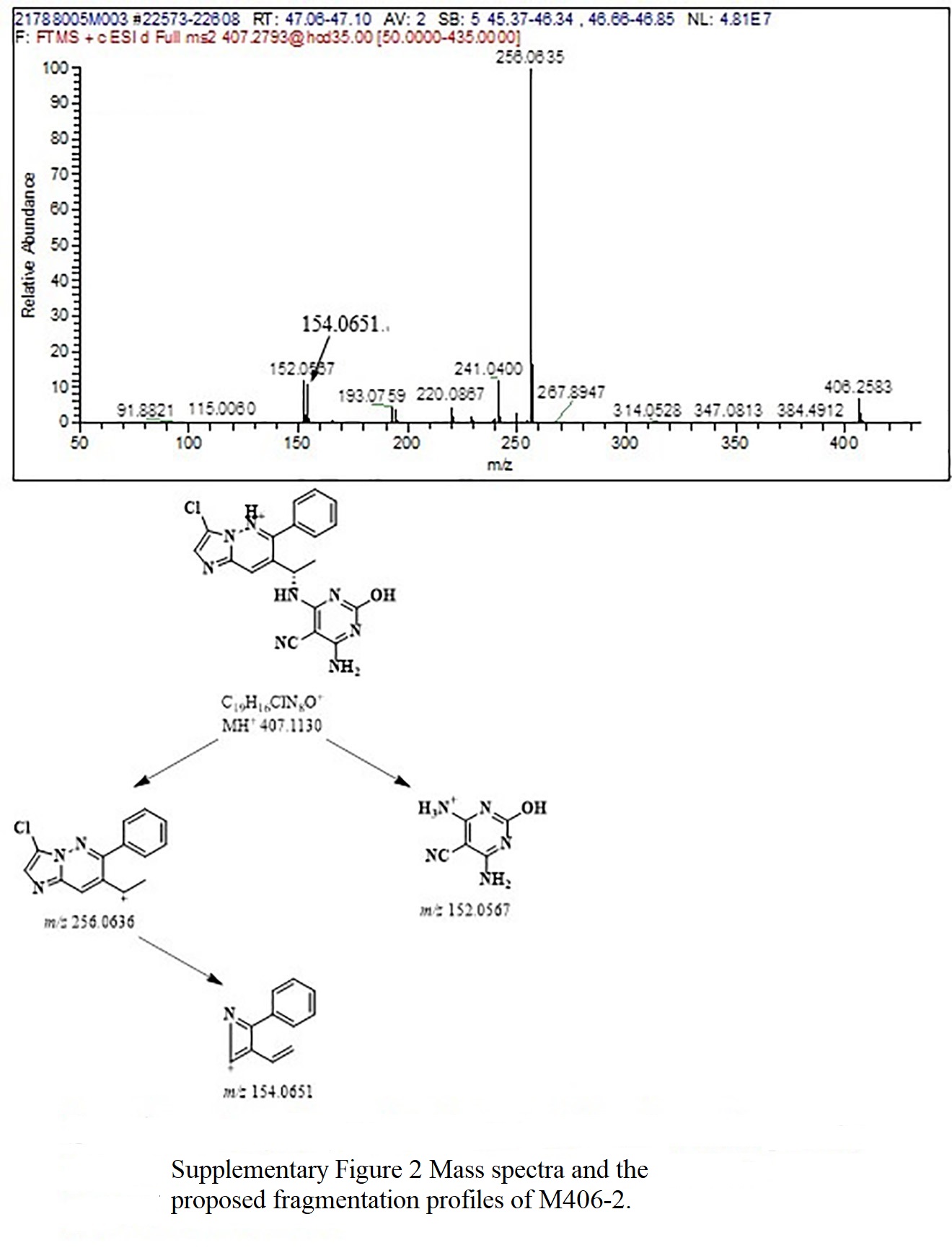

Supplement: Supplementary file 8 [file Image2.JPEG]

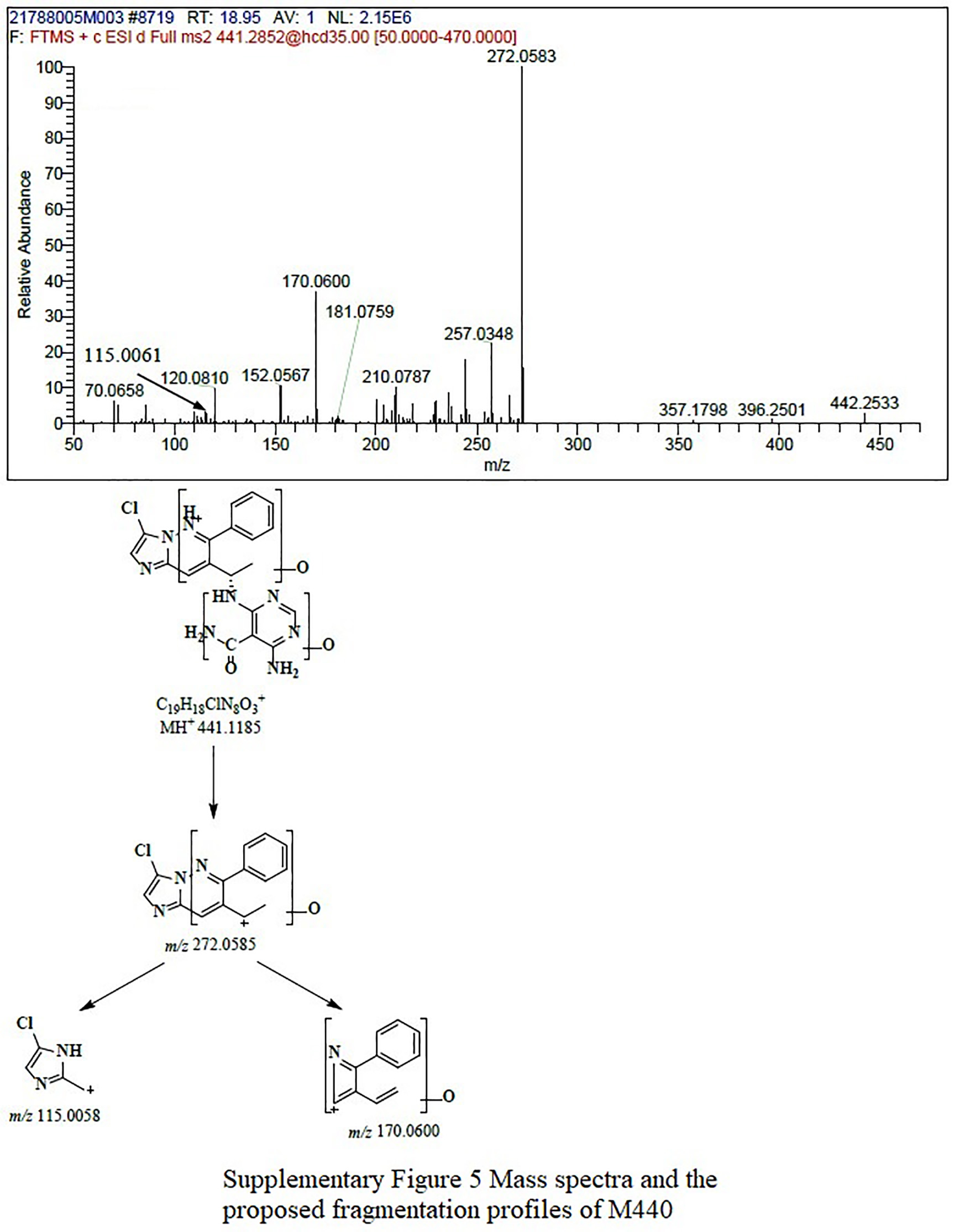

Supplement: Supplementary file 9 [file Image5.JPEG]

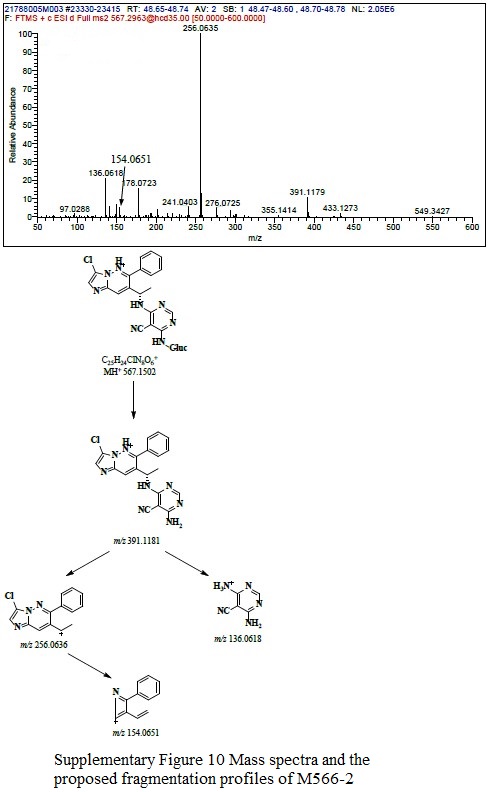

Supplement: Supplementary file 10 [file Image10.JPEG]

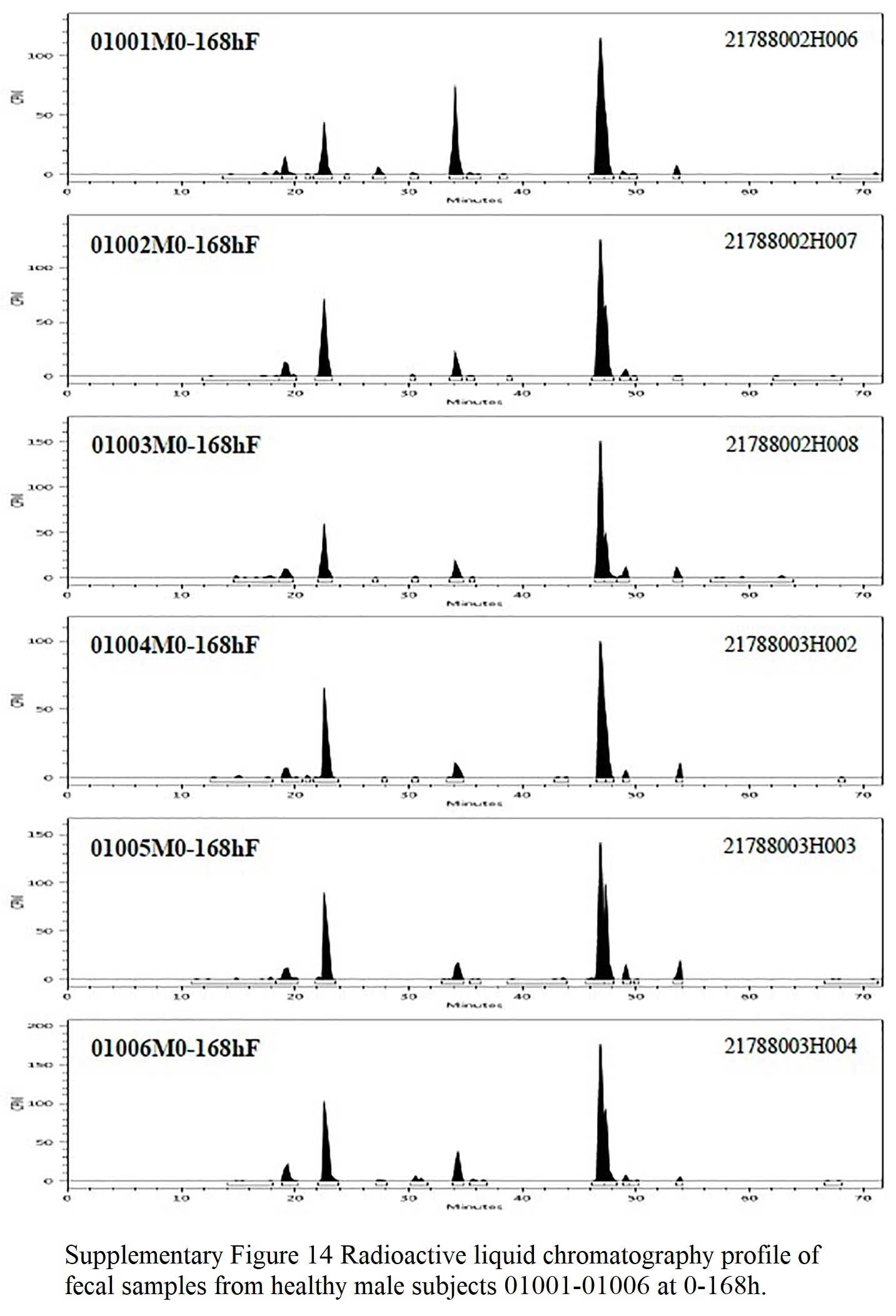

Supplement: Supplementary file 11 [file Image14.JPEG]

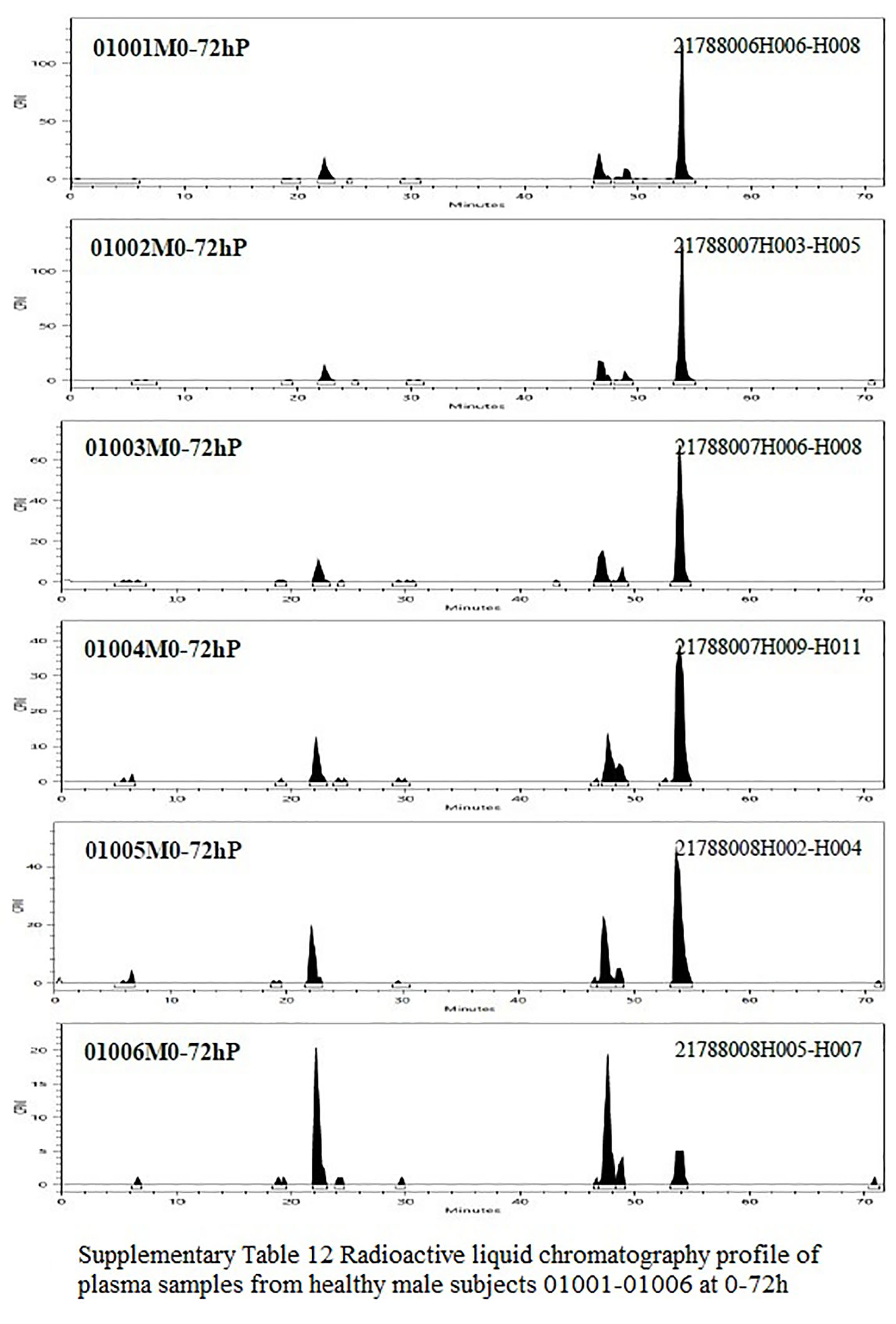

Supplement: Supplementary file 12 [file Image12.JPEG]

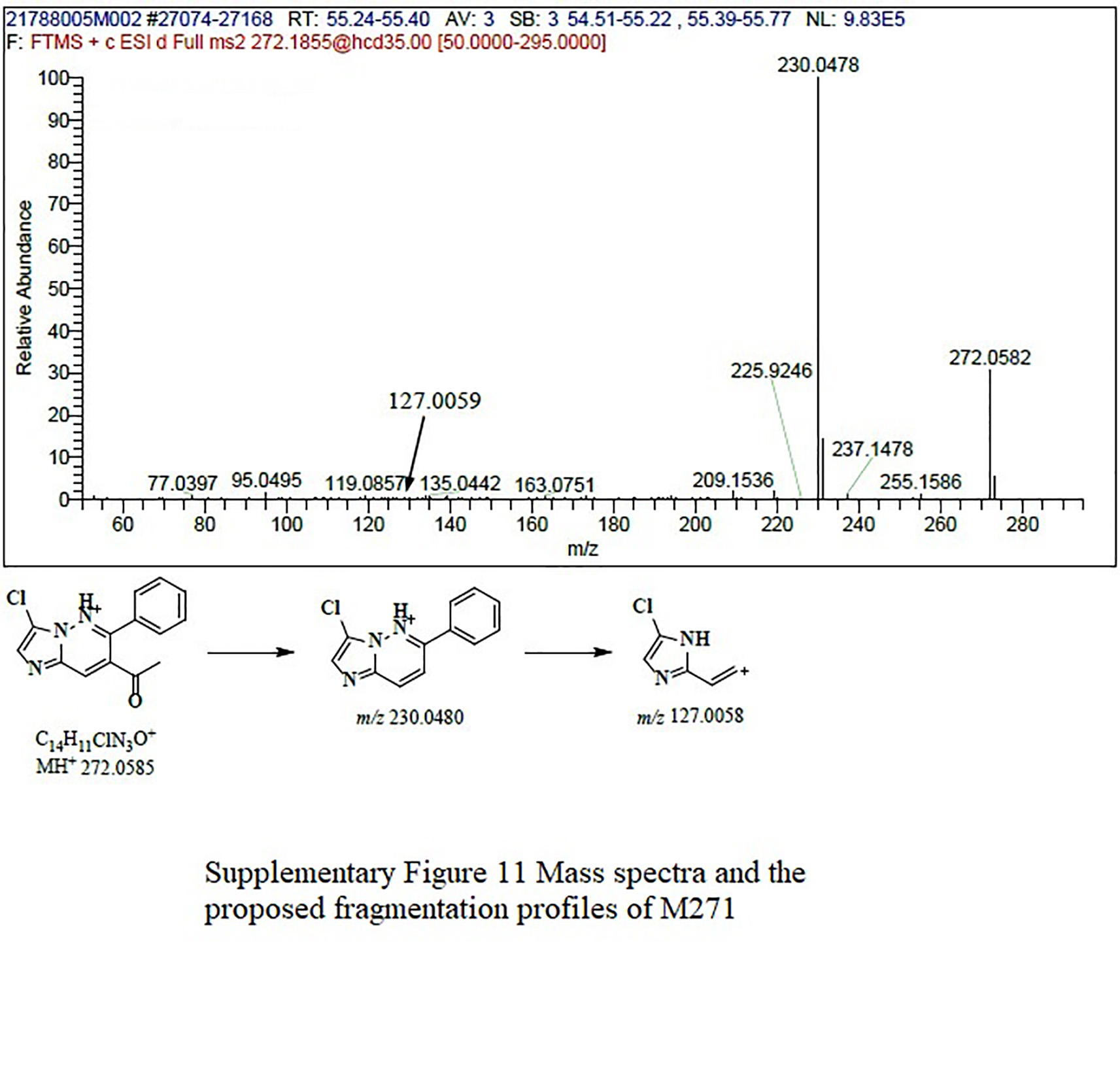

Supplement: Supplementary file 13 [file Image11.JPEG]

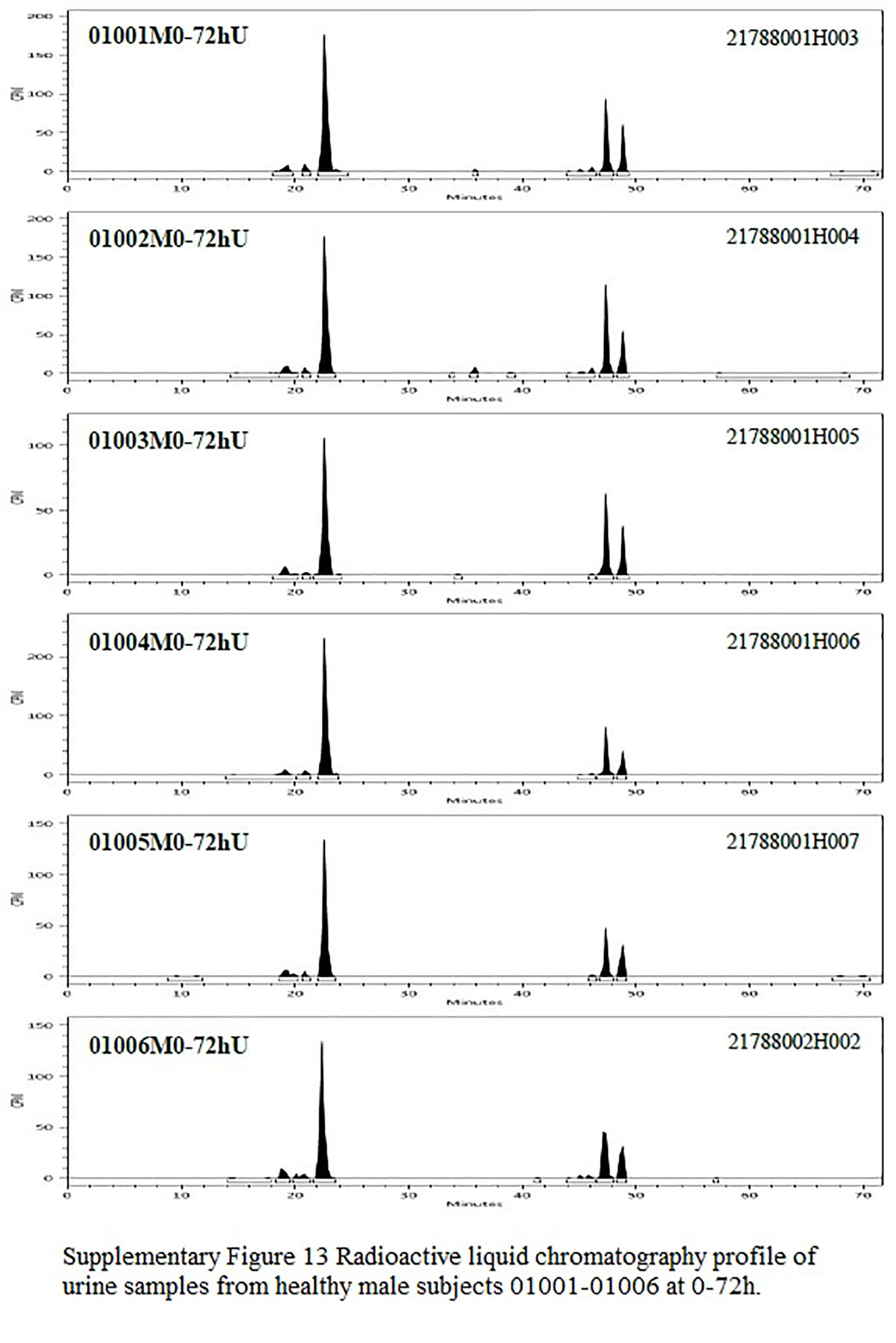

Supplement: Supplementary file 16 [file Image13.JPEG]

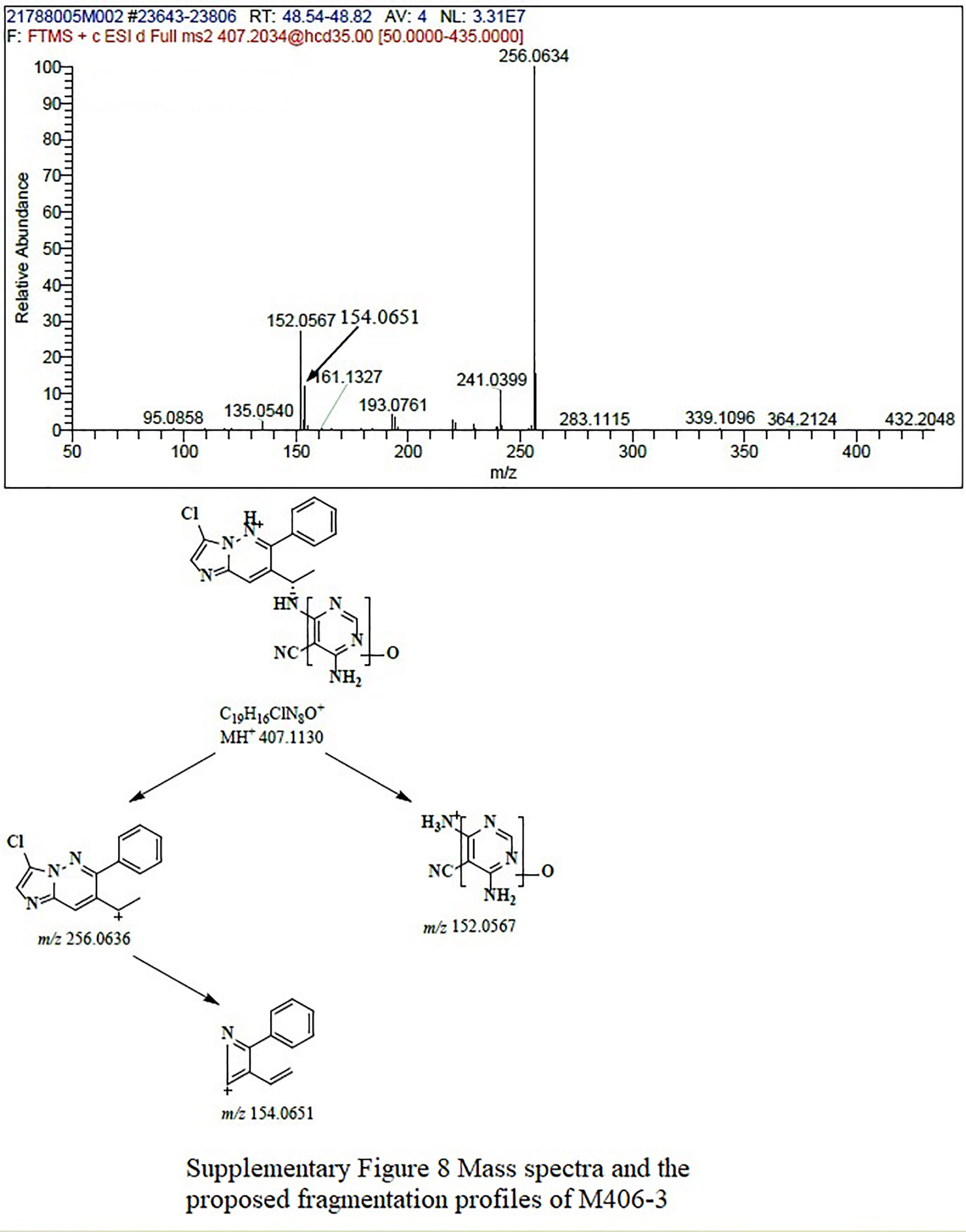

Supplement: Supplementary file 17 [file Image8.JPEG]

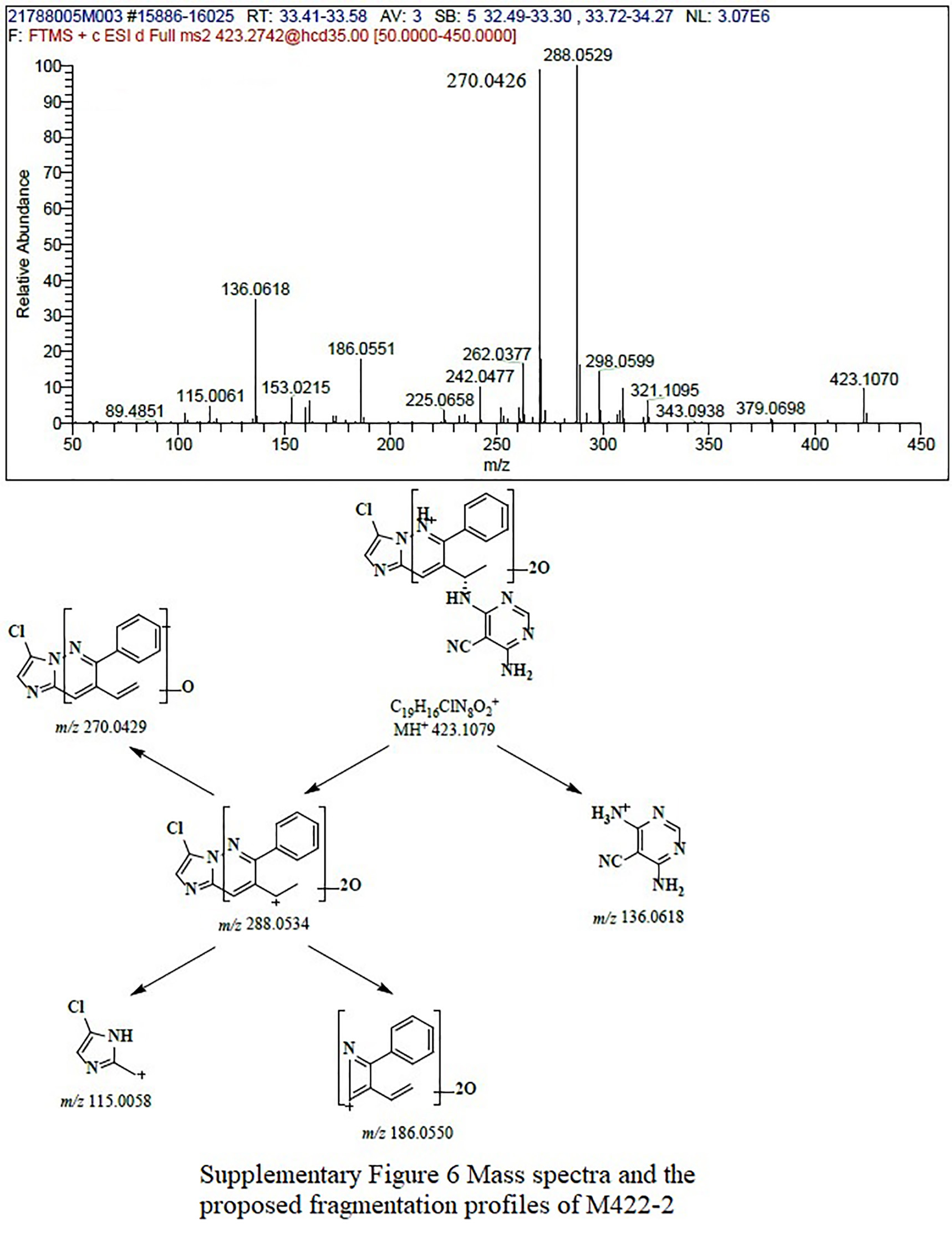

Supplement: Supplementary file 18 [file Image6.JPEG]
