# Supplementary material for: Mass balance, metabolism, and pharmacokinetics of [14C]amdizalisib, a clinical-stage novel oral selective PI3Kδ inhibitor for the treatment of non-hodgkin’s lymphoma, in healthy Chinese volunteers
Source: Front Pharmacol. 2024 Nov 15;15:1478234. doi: 10.3389/fphar.2024.1478234 (PMC11605291; doi:10.3389/fphar.2024.1478234)
Supplement: Supplementary file 3 [file Table1.docx]

Supplementary Table 1 Percentage of Total Radioactive Exposure (%AUC) of [^14^C] Amdizalisib and Its Metabolites in AUC-Pooled Plasma Samples for Individual Subjects

| Metabolites | Retention time (min) | %AUC | | | | | | Mean | SD |
| --- | --- | --- | --- | --- | --- | --- | --- | --- | --- |
|  |  | 01001 | 01002 | 01003 | 01004 | 01005 | 01006 |  |  |
| M424 | 22.1-22.4 | 13.48 | 9.89 | 12.32 | 13.44 | 15.72 | 35.14 | 16.67 | 9.25 |
| M436 | 46.6 | ND | ND | ND | 0.38 | 0.61 | 0.63 | 0.27 | 0.31 |
| M406-1 | 46.6 | ND | ND | ND | 0.16 | 0.26 | 0.27 | 0.12 | 0.13 |
| M406-2 | 46.6-47.6 | 16.31 | 16.12 | 20.20 | 16.67 | 20.09 | 36.04 | 20.91 | 7.64 |
| M406-3 | 48.6-48.9 | 8.16 | 5.50 | 5.42 | 5.91 | 4.37 | 6.31 | 5.95 | 1.26 |
| [^14^C]Amdizalisib | 53.6-53.9 | 57.45 | 65.57 | 56.65 | 59.14 | 54.59 | 15.32 | 51.45 | 18.1 |
