# Supplementary material for: Mass balance, metabolism, and pharmacokinetics of [14C]amdizalisib, a clinical-stage novel oral selective PI3Kδ inhibitor for the treatment of non-hodgkin’s lymphoma, in healthy Chinese volunteers
Source: Front Pharmacol. 2024 Nov 15;15:1478234. doi: 10.3389/fphar.2024.1478234 (PMC11605291; doi:10.3389/fphar.2024.1478234)
Supplement: Supplementary file 14 [file Table2.docx]

Supplementary Table 2 Percentage of Dose (%Dose) of [^14^C] Amdizalisib and Its Metabolites in Pooled Urine Samples for Individual Subjects

| Metabolites | Retention time (min) | %Dose | | | | | | Mean | SD |
| --- | --- | --- | --- | --- | --- | --- | --- | --- | --- |
|  |  | 01001 | 01002 | 01003 | 01004 | 01005 | 01006 |  |  |
| M527 | 18.9-19.4 | 1.41 | 1.53 | 1.49 | 1.69 | 2.09 | 1.49 | 1.62 | 0.250 |
| M424 | 22.4-22.6 | 22.94 | 18.19 | 18.99 | 26.46 | 21.80 | 17.70 | 21.01 | 3.38 |
| M422-2 | 33.9-34.4 | ND | 0.07 | 0.12 | ND | ND | ND | 0.03 | 0.0515 |
| M436 | 45.1-46.1 | 0.70 | 0.73 | 0.12 | 0.31 | 0.33 | 0.66 | 0.48 | 0.255 |
| M406-2 | 47.1-47.4 | 8.77 | 9.03 | 8.92 | 7.29 | 6.50 | 7.98 | 8.08 | 1.02 |
| M406-3 | 48.9 | 4.98 | 4.35 | 4.85 | 3.77 | 4.88 | 4.33 | 4.53 | 0.464 |
| M566-2 | 48.9 | 0.19 | 0.16 | 0.18 | 0.14 | 0.18 | 0.16 | 0.17 | 0.0183 |
