# Supplementary material for: Mass balance, metabolism, and pharmacokinetics of [14C]amdizalisib, a clinical-stage novel oral selective PI3Kδ inhibitor for the treatment of non-hodgkin’s lymphoma, in healthy Chinese volunteers
Source: Front Pharmacol. 2024 Nov 15;15:1478234. doi: 10.3389/fphar.2024.1478234 (PMC11605291; doi:10.3389/fphar.2024.1478234)
Supplement: Supplementary file 15 [file Table3.docx]

Supplementary Table 3 Percentage of Dose (%Dose) of [^14^C] Amdizalisib and Its Metabolites in Pooled Fecal Samples for Individual Subjects

| Metabolites | Retention time (min) | %Dose | | | | | | Mean | SD |
| --- | --- | --- | --- | --- | --- | --- | --- | --- | --- |
|  |  | 01001 | 01002 | 01003 | 01004 | 01005 | 01006 |  |  |
| M440 | 19.1-19.4 | 2.18 | 3.42 | 3.44 | 2.44 | 3.37 | 4.30 | 3.19 | 0.77 |
| M424 | 22.6 | 7.64 | 16.47 | 13.33 | 15.66 | 15.71 | 16.72 | 14.26 | 3.46 |
| M422-2 | 34.1-34.4 | 12.66 | 4.31 | 4.40 | 3.50 | 3.69 | 5.92 | 5.75 | 3.49 |
| M436 | 46.9 | 16.72 | 18.13 | 19.78 | 21.53 | 14.10 | 15.91 | 17.70 | 2.69 |
| M406-1 | 46.9 | 4.78 | 5.18 | 5.65 | 6.15 | 4.03 | 4.54 | 5.06 | 0.77 |
| M406-2 | 47.1-47.4 | 9.93 | 13.05 | 10.17 | 5.32 | 14.97 | 14.33 | 11.30 | 3.60 |
| M408 | 48.9-49.1 | 0.40 | 0.91 | 2.11 | 0.69 | 1.53 | 0.60 | 1.04 | 0.65 |
| M566-2 | 48.9-49.1 | 0.04 | 0.10 | 0.23 | 0.08 | 0.16 | 0.07 | 0.11 | 0.07 |
| [^14^C]Amdizalisib | 53.6-53.9 | 0.77 | 0.25 | 1.93 | 1.52 | 2.32 | 0.48 | 1.21 | 0.84 |
